# Glucocorticoid receptors are required effectors of TGFβ1-induced p38 MAPK signaling to advanced cancer phenotypes in triple-negative breast cancer

**DOI:** 10.1186/s13058-020-01277-8

**Published:** 2020-05-01

**Authors:** Carlos Perez Kerkvliet, Amy R. Dwyer, Caroline H. Diep, Robert H. Oakley, Christopher Liddle, John A. Cidlowski, Carol A. Lange

**Affiliations:** 1 Departments of Medicine (Division of Hematology, Oncology, and Transplantation) and Pharmacology, University of Minnesota Masonic Cancer Center, Delivery Code 2812 Cancer and Cardiovascular Research Building; Suite 3-126 2231 6th St SE, Minneapolis, MN 55455 USA; 2 Department of Health and Human Services, Laboratory of Signal Transduction, National Institute of Environmental Health Sciences, National Institutes of Health, Research Triangle Park, NC 27709 USA; 3 Storr Liver Centre, The Westmead Institute for Medical Research and Westmead Hospital, University of Sydney, Darlington, NSW 2006 Australia

**Keywords:** Glucocorticoid receptor, Transcription, Cytokines, TGFβ1, Breast cancer, Migration, Phosphorylation, p38 MAP kinase, MAP3K5/ASK1, 14-3-3ζ, Cellular stress

## Abstract

**Background:**

Altered signaling pathways typify breast cancer and serve as direct inputs to steroid hormone receptor *sensors*. We previously reported that phospho-Ser134-GR (pS134-GR) species are elevated in triple-negative breast cancer (TNBC) and cooperate with hypoxia-inducible factors, providing a novel avenue for activation of GR in response to local or cellular stress.

**Methods:**

We probed GR regulation by factors (cytokines, growth factors) that are rich within the tumor microenvironment (TME). TNBC cells harboring endogenous wild-type (wt) or S134A-GR species were created by CRISPR/Cas knock-in and subjected to transwell migration, invasion, soft-agar colony formation, and tumorsphere assays. RNA-seq was employed to identify pS134-GR target genes that are regulated both basally (intrinsic) or by TGFβ1 in the absence of exogenously added GR ligands. Regulation of selected basal and TGFβ1-induced pS134-GR target genes was validated by qRT-PCR and chromatin immunoprecipitation assays. Bioinformatics tools were used to probe public data sets for expression of pS134-GR 24-gene signatures.

**Results:**

In the absence of GR ligands, GR is transcriptionally activated via p38-dependent phosphorylation of Ser134 as a mechanism of homeostatic stress-sensing and regulated upon exposure of TNBC cells to TME-derived agents. The ligand-independent pS134-GR transcriptome encompasses TGFβ1 and MAPK signaling gene sets associated with TNBC cell survival and migration/invasion. Accordingly, pS134-GR was essential for TNBC cell anchorage-independent growth in soft-agar, migration, invasion, and tumorsphere formation, an in vitro readout of cancer stemness properties. Both pS134-GR and expression of the MAPK-scaffolding molecule 14-3-3ζ were essential for a functionally intact p38 MAPK signaling pathway downstream of MAP3K5/ASK1, indicative of a feedforward signaling loop wherein self-perpetuated GR phosphorylation enables cancer cell autonomy. A 24-gene pS134-GR-dependent signature induced by TGFβ1 predicts shortened overall survival in breast cancer patients.

**Conclusions:**

Phospho-S134-GR is a critical downstream effector of p38 MAPK signaling and TNBC migration/invasion, survival, and stemness properties. Our studies define a ligand-independent role for GR as a homeostatic “sensor” of intrinsic stimuli as well as extrinsic factors rich within the TME (TGFβ1) that enable potent activation of the p38 MAPK stress-sensing pathway and nominate pS134-GR as a therapeutic target in aggressive TNBC.

**Electronic supplementary material:**

The online version of this article (10.1186/s13058-020-01277-8) contains supplementary material, which is available to authorized users.

## Background

Breast cancer (BC) accounts for ~ 15% of cancer-related death in American women [1]. The primary method of classifying breast cancer relies on the presence or absence of estrogen receptor (ER), the ER target gene progesterone receptor (PR), and amplification of the human epidermal growth factor receptor 2 (HER2) [2, 3]. These molecular markers in part dictate the treatment regimen that BC patients receive [4]. When these receptors are present, targeted therapies are used to decrease recurrence rate and improve survival (e.g., tamoxifen inhibits ER and trastuzumab/pertuzumab targets HER2 [5]). However, triple-negative breast cancers (TNBC), which account for ~ 15% of all breast cancer patients, lack these receptors and are thus defined by this feature [6]. Although TNBC is intensely studied, molecular targeted therapies are still largely unavailable for TNBC patients, who suffer from higher disease recurrence, more frequent metastasis, and a worse prognosis relative to patients with other BC subtypes [6]. Thus, appropriate biomarkers of driver pathways and new therapeutic targets are urgently needed.

Up to 40% of TNBC tumors express elevated glucocorticoid receptor (GR) levels [7–11]. GR is a ligand-activated (cortisol/dexamethasone [Dex]) transcription factor member of the steroid hormone receptor (SR) superfamily whose expression is associated with resistance to chemotherapy and metastatic recurrence of TNBC [7–11]. Recently, a GR gene signature was employed to stratify patients by prognosis; patients who expressed high/low levels of the 74 GR target gene mRNAs experienced shortened disease-free survival [12]. As with other SRs, GR is subject to posttranslational modifications that impact its function. Similar to ER [13] and both PR isoforms [14], phosphorylation events dramatically alter GR target gene selection [15]. We showed previously that phosphorylation of GR on Serine 134 is elevated in TNBC relative to other breast cancer subtypes [16]; this p38 MAPK-dependent event was insensitive to GR ligands, but induced in response to cellular stress, including ROS/H_2_O_2_, hypoxia, and nutrient starvation [17], as well as loss of attachment (i.e., cell suspension) and stress-inducing chemotherapies [16] such as paclitaxel (Taxol), a taxane microtubule stabilizing drug routinely used as adjuvant or neoadjuvant chemotherapy in TNBC patients [16, 18]. Upon ligand (i.e., Dex, cortisol) binding, phospho-Ser134 GR (pS134-GR) upregulated Hypoxia-Inducible Factor 2 (HIF2), AhR (Aryl Hydrocarbon Receptor), and Breast Tumor Kinase (i.e., Brk encoded by the *PTK6* gene). These ligand-dependent pS134-GR target genes are known mediators of pro-survival and metastasis in TNBC [16, 17].

The metastatic cascade is a multi-step process that is regulated by both intrinsic and extrinsic factors [19–21]. Cancer cells must detach from the primary tumor mass, migrate and invade through surrounding local tissues, intravasate to the lymph or blood vessels, survive in the circulation as individual cells or as collective cell clusters, and extravasate from the vessels to the target tissue in order to finally colonize a new metastatic lesion [21]. The tumor microenvironment (TME) contributes to many of these steps [22]. Stromal cells within the primary tumor express cytokines and growth factors that modulate the invasive potential of breast epithelial carcinoma cells. Notably, Transforming Growth Factor β1 (TGFβ1) promotes cancer cell migration/invasion and is associated with poor outcome in TNBC patients [23]. Inhibition of TGFβ signaling in pre-clinical models attenuated cell migration and blocked epithelial to mesenchymal transition (EMT) in TNBC [24, 25]. TGFβ1 activates the TGFβ Ser/Thr kinase receptors (types I and II), resulting in phosphorylation and activation of SMAD transcription factors [26]. Phospho-SMADs enter the nucleus and induce the expression of genes that promote both migration and invasion [26]. Importantly, SMAD-independent pathways are also regulated by TGFβ; stress-activated protein kinases, including p38 MAPKs, are rapidly activated downstream of TGFβ receptors [27]. Emerging data support a role for p38 MAPK signaling in advanced breast cancer biology. For example, p38 MAPK is essential for promoting lung metastasis in response to TGFβ [28]. Namely, SRs, including GR, PR, and androgen receptor (AR), are important substrates for MAPKs [17, 29, 30].

Herein, we sought to better understand the intersection of GR-driven actions with the TGFβ1 signaling pathway in TNBC. Although molecular cooperation between GR and TGFβ1 has been studied in other fields (e.g., immunology), its relevance to TNBC is under-explored. Herein, we show that TGFβ1 induces robust p38 MAPK-dependent GR Ser134 phosphorylation; this event is critical for TGFβ1-induced TNBC cell anchorage-independent growth and migration. TGFβ1-mediated migration requires pS134-GR, but not exogenously added GR ligands and is blocked by GR antagonists. Remarkably, we find that pS134-GR is essential for the expression of MAP3K5, a key upstream regulator of MEK3/6 and p38 MAPK required for pathway activation. Finally, a novel gene signature composed of 24 pS134-GR-regulated transcripts predicts rapid disease progression in BC patients. We conclude that pS134-GR is both a major effector of the p38 MAPK signaling pathway downstream of the TME-derived factor TGFβ1 and required for a functionally intact p38 MAPK module (i.e., MAP3K5 - MEK3/6 - p38 MAPK). The amplification of p38 MAPK signaling by pS134-GR represents a feedforward signaling loop that may be disrupted to halt TNBC progression. Phospho-GR species represent novel biomarkers for co-targeting GR and p38 MAPK in TNBC.

## Materials and methods

### Cell lines and culture conditions

Parental (unmodified) MDA-MB-231 cells are a gift from Dr. Ronald Wegener and were maintained in 10% FBS and 1% P/S. Cells that were used for generation of CRISPR/Cas cell line were authenticated on April 27, 2017, by the University of Arizona Genetics Core and were compared to the ATCC short-tandem repeat (STR) database. Additionally, cells tested negative for Mycoplasma. U2OS cell lines were obtained from Dr. John Cidlowski. These cells were grown in 10% FBS, 1% P/S, 1% glutamax, 0.2 mg/mL Hygromycin, and 200 μg G418. The TNBC cell lines: Hs578T, MDA-MB-468, and HCI-10 cells were maintained in 10% FBS and 1% P/S. All of these cell lines tested negative for Mycoplasma.

### Materials and reagents

All materials and reagents are listed in Supplementary Table 5.

### Generation of MDA-MB-231 NR3C1 (GR) S134A knock-in cells (CRISPR)

#### Generation of customized guide RNA expression construct

In order to genetically modify MDA-MB-231 cells to express NR3C1 containing an S134A mutation, CRISPR/Cas9-mediated gene targeting was implemented. This was accomplished using an HDR donor vector along with a CRISPR/Cas9-GFP expression vector; an anti-sense gRNA sequence (NR3C1-S134A-AS1-sgRNA) targeting the coding sequence of NR3C1 was cloned into a CRISPR/Cas9-GFP expression vector (PX458). For the generation of the CRIPSR/Cas9-GFP vector, a sense oligo 5′-CACCGCTTGGGGTTCTCTGGAACAC-3′ and an anti-sense oligo 5′-AAACGTGTTCCAGAGAACCCCAAGC-3′, containing four base-pair overhangs compatible with PX458 BbsI restriction enzyme digestion sites, were annealed and ligated into PX458. In addition, a second anti-sense sgRNA sequence (NR3C1-S134A-AS2-sgRNA) 5′-CAGTGGATGCTGAACTCTTG-3′ was incorporated in an identical way into a separate PX458 plasmid. Correct incorporation of both sgRNA sequences was confirmed by Sanger sequencing (Genewiz).

#### Generation of a dsDNA plasmid donor for the incorporation of S134A mutation in the coding sequence of *NR3C1*

The donor vector used to incorporate the S134A mutation into the *NR3C1* (glucocorticoid receptor) coding region was generated using a golden gate strategy. Briefly, pAAV-GG-MCS-SEPT-Neo, a kind gift from the Hendrickson Laboratory at the University of Minnesota, was used to incorporate a 2100-bp sequence that spans the coding sequence of NR3C1 and contains the S134A (AGT – GCT) mutation as well as a single silent mutation within the sgRNA sequence to interrupt the protospacer adjacent motif (PAM) for both sgRNAs (i.e., to prevent repeated Cas9 cleavage after successful incorporation of the donor by homology directed repair). Conveniently, the AGT to GCT S134A mutation also generates a unique PvuII restriction enzyme site (CAGCTG) that was used for screening for desired clones. The left homology arm was generated using primers LArmF 5′-GACGCTCTTCACCGGGCACATCCAGTCAGAAGTATGGGT-3′ and LArmR 5′-GACGCTCTTCTCTCTGGAACAgctGTCGACCTATTGAG-3′ while the right homology arm was generated using primers RArmF 5′-GACGCTCTTCCGAGAAtCCCAAGAGTTCAGCATCCAC and RArmR 5′-GACGCTCTTCGATGAATAAAATCCTCACCGTTGGCCAATGG-3′. The left and right homology arms were cloned into pAAV-GG-MCS-SEPT-Neo using BspQI golden gate cloning.

#### MDA-MB-231 transfections and screening for *NR3C1* S134A knock-in clones

The PX458-NR3C1-S134A-AS1-sgRNA or PX458-NR3C1-S134A-AS2-sgRNA plasmids along with the pAAV-GG-NR3C1-S134A donor vector were transfected into MDA-MB-231 cells using Lipofectamine 3000 (Invitrogen) and allowed to recover for at least 2 days. GFP-positive cells were then collected by FACS sorting, expanded, and sub-cloned by limited dilution into 96-well plates. Single-cell clones were identified and expanded further into 24-well plates for genomic DNA collection and PCR screening. In order to identify CRISPR/Cas9 edited cells, two primers that span the CRISPR/Cas9 cut sites for NR3C1 S134A AS1 and AS2 NR3C1_S134A_ScrF2 5′- GGCTGTCGCTTCTCAATCA-3′ and NR3C1_S134A_ScrR2 5′- GGACTCTCATTCGTCTCTTTACC-3′ were used to produce a 492-bp amplicon by PCR. Clones that had correctly incorporated the S134A mutation were identified by performing PvuII restriction enzyme digestion on the resulting amplicons. Amplicons that exhibited PvuII cleavage by gel electrophoresis were sequenced using the NR3C1_S134A_ScrF2 primer.

Two independent clones were identified as having incorporated the intended S134A mutation as well as the PAM mutation in a bi-allelic fashion. To control for off-target cutting by CRISPR/Cas9 during the generation of these clones, a non-targeted clone, 34A, was also recovered from the population (wt-GR cells).

### Generation of MDA-MB-231 shcontrol and sh14-3-3ζ cells

Stable sh14-3-3ζ (TRCN0000029404 and TRCN0000029406) were generated by transducing MDA-MB-231 models with CMV-Neo lentiviral vectors containing target gene shRNA sequences (MISSION TRC library - Sigma). Pools were selected and maintained through culture with 0.75 mg/mL of G418 sulfate (Corning).

### Scratch wound migration assay

Cells were plated in a six-well plate at a density of 1.5 × 10^5^ cells to achieve monolayer growth at 100% confluency. Pretreatment (hormone, growth factor) was performed at various times before the scratch was created, as indicated. Experiments that required hormone (Dex), growth factor (Hepatocyte Growth Factor—HGF), or cytokine (Transforming Growth Factor Beta β1 – TGFβ1) treatment, cells were starved for 18–24 h in Modified Improved Minimum Essential Media (IMEM) containing 1% DCC. A 200-μL sterile pipette tip was used to create each scratch. After scratching was performed, cells were maintained in 37 °C, 5% CO_2_ with their respective treatments (see legends). Three independent wells per group were imaged by taking three images of each well at × 10 magnification. ImageJ was used for quantification of the area of the wound, and three biological replicates were used per cell line. Data are shown as a representative of three experimental replicates.

### Cell proliferation assay

Proliferation of wt-GR and S134A-GR MDA-MB-231 cells in basal medium was measured via MTT assays ((3-[4,5-dimethylthiazol-2-yl]-2,5-diphenyltetrazolium bromide). Using 24-well plates, 2.5 × 10^4^ cells/well were plated. At days 0, 1, 3, and 7, cell proliferation was determined. Sixty microliters of MTT was added per well for a final concentration of 5 mg/mL. Plate was incubated at 37 °C for 3 h. At this point, the medium was removed and solubilization solution (90% v/v dimethyl sulfoxide (DMSO)/PBS) was added to lyse the cells. Absorbance was measured in plate reader at 650 and 570 nm. The 650-nm measurements were subtracted from the 570-nm measurements. Sample means were normalized to day 0 and plot ± standard deviation (SD) using a linear plot.

### Western blot

Cells were plated, and for experiments that required hormone (Dex), growth factor (HGF), or cytokine (TGFβ1) treatment, cells were starved for 18–24 h in Modified Improved Minimum Essential Media (IMEM) containing 1% DCC. Whole-cell lysates were prepared using RIPA lite supplemented with 1 mmol/L PMSF, 5 mmol/L NaF, 0.05 mmol/L Na_3_VO_4_, 25 mmol/L beta glycerophosphate (BGP), 20 μg/mL aprotinin, 1 complete mini tablet of protease inhibitors (Roche), and 1 tablet of PhosSTOP (Roche). Electrophoresis in an SDS-PAGE gel was used to resolve and separate the proteins. Fifty micrograms of protein per lane was loaded. After electrophoresis, proteins were transferred to polyvinylidene difluoride membranes and probed with the following antibodies: pGR (1:750 - custom made, Pierce Biotechnology), GR (1:1000 - Santa Cruz Biotechnology, sc-1003), p-p38 (1:1000 - Cell Signaling Technology, 4511p), p38 (1:1000 - Cell Signaling Technology, 9212), YWHAZ (14-3-3ζ – 1:3000 – Millipore, AB9746), MAP3K5/ASK1 (1:1000 – Santa Cruz Biotechnology, sc5294), pERK1/2 (1:1000 – Cell Signaling Technology, 9101S), ERK1/2 (1:1000 – Cell Signaling Technology, 9102S), pJNK(1:1000 – Cell Signaling Technology, 9251S), and JNK (1:1000 – Cell Signaling Technology, 9252S) in 1% milk. For densitometric calculations, Fiji software version 2.0 was used to quantify bands representing total and phosphorylated proteins (GR, pS134-GR, p38, p-p38). All samples were normalized to respective controls and fold-change calculated relative to the vehicle condition. Densitometric data were collected for at least two to three independent experiments, and relative values were plotted as the mean ± SEM; the one-way ANOVA post hoc Fisher test was utilized to assess statistical significance.

### Cell migration and invasion transwell assays

Cell migration was measured using 8-μm transwell Boyden Chamber inserts (Corning). Cells were pretreated as indicated by the figure legends. After pretreatment, cells were trypsinized and plated at a density of 5 × 10^4^ cells in the upper chamber of the transwell system in IMEM media. Chemoattractant agents that were added to the lower-well are indicated in the figure legends in addition to 1% DCC. Cells were incubated for the time indicated in figure legends and maintained in 37 °C, 5% CO_2_. Cellular invasion assays were done in a similar fashion with the exception that Matrigel (Corning) transwell inserts were utilized; Matrigel inserts were placed for at least 2 h in the incubator before plating the cells. Transwell inserts were imaged using a light microscope. Four pictures per transwell were taken at × 10 magnification. Three biological replicates were used in each condition and data are shown as representative of three experimental replicates. Results are reported as the mean percentage of migration for each well relative to wt-GR ± SD.

### Colony formation assay

Six-well plates were prepared, and a base agarose layer (Invitrogen) was created in the surface of the wells. Cells were trypsinized and counted, and 4 × 10^4^ cells were plated in the top agarose layer in triplicate per treatment condition (indicated in figure legends). Plates were kept at 4 °C for 10 min to allow the agar to solidify. Incubation of plates occurred at 37 °C and 5% CO_2_ for 15 days. To quantify colonies, 1 mL of PBS containing 4% formaldehyde and 0.005% crystal violet was added to each well. Cells were incubated for 1 h at room temperature. Four images were taken in each well. Results are reported as colonies counted per field and using mean ± SD.

### Tumorsphere assay

A single-cell solution was obtained after enzymatic dissociation in 0.25% trypsin-EDTA and strained through a 40-μm sieve (BD Falcon). Cells were plated in ultra-low attachment plates (Corning) at 1 × 10^3^ cells per well and grown in a serum-free DMEM/F12 phenol-free medium (Corning) containing 1% methylcellulose (Sigma Aldrich), 1% B27 proprietary supplement (Invitrogen), 1% penicillin-streptomycin, 20 ng/ml EGF (Sigma Aldrich), 20 ng/ml basic-FGF (Gibco), and 10 μg/ml heparin (Sigma Aldrich). After 5–7 days, generate secondary tumorspheres were generated; primary spheres were collected and dissociated enzymatically in 0.25% trypsin-EDTA. Single cells were plated as described in conditioned media, which consisted of a 1:1 mixture of DMEM/F12 tumorsphere media (as above) and media from cultured parental cells. The tumorspheres were allowed to grow for 5–7 days before manual counting. Data are presented as the average ± SD of two independent measurements.

### RNA expression analysis for METABRIC samples

Molecular Taxonomy of Breast Cancer International Consortium (METABRIC) data (via cBioPortal – http://www.cbioportal.org/) were obtained for patients who had the three-gene classifier and microarray data available for MAP3K5 and 14-3-3ζ [31–33]. After that, we log2 transformed all of the mRNA expression values. Boxplot was utilized to plot the data and compare the expression across groups. Statistical significance was calculated based on one-way ANOVA and Tukey post hoc. All data associated to this analysis are in Supplementary File 1 and 2.

### RNA library preparation and sequencing

MDA-MB-231 expressing either wt-GR or S134A-GR were serum starved in IMEM containing 1% DCC for 18 h. Cells were treated with vehicle treatment or either 10 ng/mL of TGFβ1 for 6 h. Total RNA was extracted using the Qiagen RNAeasy kit. RNA quality and concentration were assessed by nanodrop. A TruSeq RNA kit was utilized to prepare and generate the mRNA libraries that were sequenced on the Illumina HiSeq2500 in a 75-base paired-end mode. Forty million reads were sequenced on average per sample.

### RNA-seq data processing

Quality of raw RNA-seq sequences for each sample was assessed with FastQC. Trimmommatic was used to remove low-quality bases and trim adapters. FastQC was used again on each filtered FASTQ files to generate a sequence quality plot. Alignment to the hg38 genome was performed using HISAT2 [34]. Abundance of transcript was estimated with the featureCounts program in the SubRead package. DESeq2 (Version 1.22.2) was used to evaluate differential expression across groups and generate principal component analysis (PCA) plots [35]. For analyses of data using the generalized linear model framework, we utilized the EdgeR package (v3.26.8). Generation of heatmaps was performed with the pheatmap package (Version 1.0.12) using log2 normalized read counts from DESeq2 analysis. The package that was used to perform the gene set enrichment analysis was fgsea (Version 1.8.0). Ingenuity Pathway Analysis was also used to evaluate pathway significance. To do this, we uploaded the differential expression gene analysis obtained from the DESeq2 and EdgeR (Generalize Linear Model) analysis. All packages were used in the R environment (Version 3.5.2) and through R Studio (Version 1.1.383). In general, the cutoff for differential expression was an absolute log2 fold-change of 1.5 and a Benjamini-Hochberg *p*-adjusted value of 0.05. Figure legends include the criteria for gene selection.

### Quantitative RT-PCR

Quantitative real-time PCR (qRT-PCR) experiments were conducted as previously described [36]. The cDNA was generated from total RNA extracted from MDA-MB-231 cells expressing either wt-GR or S134A-GR. Cells were plated at 5 × 10^4^ cells/well in six-well plates. After their respective treatments, media was removed, and cells were washed twice with cold 1× PBS. RNA was isolated with trizol, as per the manufacturer’s protocol. The cycling conditions by qPCR were as follows: 10 min of initial denaturation at 95 °C, 10 s denaturation at 95 °C, 10 s at 60 °C for annealing of primers and extension at 72 °C for 5 s for 45 cycles. Relative target gene expression was normalized to the expression of internal control genes, either *TATA-binding protein* (*TBP*), *Actin* (beta-actin*)*, or *18S rRNA* and they are shown as the mean value of three biological replicates ± SD. The primers used were MAP3K5-F 5′-AGGTGGTACTCTTTGGTTTTCAAG-3′ and MAP3K5-R 5′-GATACTGTCTAAGGCAAACATCCAG-3′; LEFTY2-F 5′-CTGGACCTCAGGGACTATGG-3′ and LEFTY2-R 5′-TCCCCTGCAGGTCAATGTAC3’; PIK3IP1-F 5′-CCTGGTGCTACGTCAGTGG-3′ and PIK3IP1-R 5′-TCCTGGATTTCTGTCGTGAAG-3′.

### Correlation of GR (*NR3C1)* and MAP3K5 in TNBC patients

RNA-seq data available as RSEM was downloaded for patients with negative status for ER, PR, and HER2 for the TCGA breast cancer provisional dataset using cBioPortal [31, 32]. All expression data was log2 transformed for both genes. PRISM (GraphPad) was employed to plot each value and to execute the Pearson analysis of correlation. All data associated to this analysis are in Supplementary File 3.

### Chromatin immunoprecipitation assay (ChIP)

ChIP assays were performed as per the manufacturer’s instruction (ChIP-IT express – Active Motif). Following respective treatments as indicated in figure legends, 37% formaldehyde was used for fixation (5 min) and sonication was utilized to shear chromatin for 30 min. Lysates were immunoprecipitated for 4 h with the following antibodies: 2 μL of GR (sc-1003) and equal amount of rabbit IgG. QPCR was used to analyze the resulting DNA and the data is represented as a percentage of input DNA. The primers used were LEFTY2 ChIP F 5′-CCCTCTAGTGGTTACAGGAAGACTC-3′ and LEFTY2 ChIP R 5′-AAAATCTGAGAGCAACTGAAGTGAG-3′; PIK3IP1 CHIP F 5′-GTACAAGTGCCCTGATAGGATTG-3′ and PIK3IP1 CHIP R 5′-CACTTCCCAGAACTGTTTTCAAC-3′. These primers were designed based on previous ChIP-Seq studies that focus on bindings sites of the GR [37, 38].

### Survival analysis for METABRIC and TCGA (SurvExpress) samples

The METABRIC microarray expression data and survival data were downloaded for the following genes via cBioPortal – http://www.cbioportal.org/: *YWHAZ*(14-3-3ζ), *DLEU7*, *JUNB*, *LBH*, *SNAI1*, *C1orf106*, *CCL20*, *NRP2*, *PIK3IP1*, *SYT8*, *KRT16*, *NLRC3*, *LEFTY1*, *NKD1*, *KPNA7*, *LTBP3*, *MDFI*, *SYN1*, *LEFTY2*, *MYOZ1*, *GPR183*, *MATK*, *STK19*, *HRAS*, and *COL8A2* as available for subjects. First, we log2 transformed all of the mRNA expression values. In the case of the survival analysis for 14-3-3ζ (Fig. 4B), we classified patients into upper 50th percentile and bottom 50th percentile based on the median cutoff for the log2 mRNA expression of 14-3-3ζ across all patients. All data associated to this analysis are in Supplementary File 4. For our gene signature, R programming was used to calculate the average gene expression of all genes from each patient and stratify groups based on the median of the average expression into upper 50th percentile or bottom 50th percentile. Kaplan-Meier plots and logrank test was used to assess the differences in overall survival between the cohorts using PRISM (GraphPad). All data associated to this analysis are in Supplementary File 5.

SurvExpress was used to analyze survival data for the TCGA cohort. The 24 TGFβ1-induced pS134-GR-dependent gene signature (*DLEU7*, *JUNB*, *LBH*, *SNAI1*, *C1orf106*, *CCL20*, *NRP2*, *PIK3IP1*, *SYT8*, *KRT16*, *NLRC3*, *LEFTY1*, *NKD1*, *KPNA7*, *LTBP3*, *MDFI*, *SYN1*, *LEFTY2*, *MYOZ1*, *GPR183*, *MATK*, *STK19*, *HRAS*, and *COL8A2*) was utilized for the SurvExpress analysis. Data was downloaded and analyzed locally in R. PRISM (GraphPad) was used to generate the Kaplan-Meier plots. Logrank test was used to assess the differences in survival between expression cohorts. All data associated to this analysis is in Supplementary File 6.

### Co-immunoprecipitation assays

Cells were lysed in ELB lysis buffer containing the following: 50 mmol/L HEPES, 0.1% nonidet P-40 (NP-40), 250 mmol/L NaCl, 5 mmol/L EDTA, 1× complete protease inhibitors (Roche), 1× PhosSTOP (Roche), 1 mmol/L PMSF, 1 mmol/L NaF, 0.5 mmol/L Na_3_PO_4_, 25 mmol/L BGP, and 20 μg/mL aprotinin]. 1000 μg of lysate was incubated with 1 μg of specific antibodies overnight at 4 °C in a rotator. Protein G agarose was used to isolate the complexes for 1 h at 4 °C. SDS-PAGE and western blot analysis were used to analyze the immunocomplexes.

### Statistics

All statistic information is disclosed in the legends. In general, for experiments that involve two groups, one-paired Student’s t-test was performed. For experiments that involved more than two groups, one-way or two-way ANOVA were utilized. Post-hoc tests within the vehicle or control treatment was performed using Dunnett’s statistical testing. However, if comparison within different groups was required, Tukey’s post-hoc test was utilized. For densitometric analysis, one-way ANOVA and Fisher Least Significant Difference (LSD) post hoc. Statistical tests were performed using either PRISM v7/v8 and R v3.5.

## Results

### Dexamethasone promotes biphasic migration in TNBC models

**Fig. 1:**
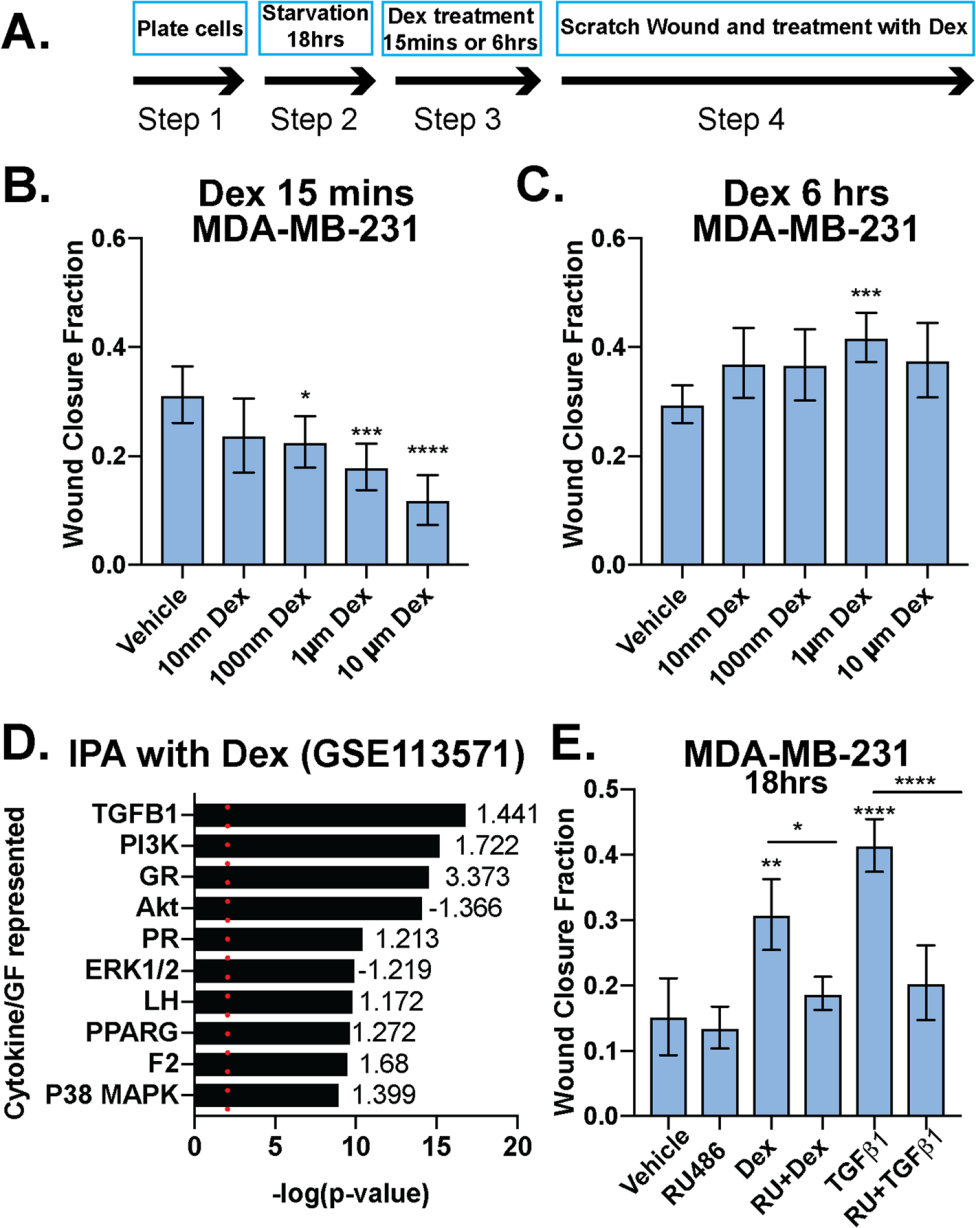
Dexamethasone either inhibits or promotes breast cancer cell migration in a time-dependent manner. **a** Schematic of protocol used for **b** and **c**. MDA-MB-231 cells were pretreated with increasing doses of Dex for either 15 min (**b**) or 6 h (**c**) and Dex-induced cell migration was measured by the degree of scratch wound closure at 18 h. The mean of three field images from each of the three biological replicates experiments is shown ± standard deviation (SD). Fraction of wound area closure of MDA-MB-231 cells was determined ImageJ. Statistical significance was assessed by one-way ANOVA and Dunnett’s post hoc for comparison within groups vs. vehicle treatment. (*, *p* < 0.05; **, *p* < 0.01; ***, *p* < 0.001; ****, *p* < 0.0001). **d** Ingenuity Pathway Analysis (IPA) for MDA-MB-231 cells treated with 100 nM Dex from GSE113571 [12]. *p* value plot shows GR-mediated activation of cancer-relevant signaling pathways and their respective *z*-scores of activation/inhibition, including the TGFβ1 and p38 MAPK pathways. The top 10 most significant pathways for cytokine/growth factor (GF) signaling are shown. Red dotted line indicates the significance value of 1.3 (*p* < .05). **e** Fraction of wound area closure of MDA-MB-231 cells treated (18 h) with vehicle control, TGFβ1 (10 ng/mL), Dex (1 μM), TGFβ1+Dex, RU486 (1 μM), RU486+TGFβ1, or RU486+Dex. The mean of three fields from each of the three biological replicate experiments is shown ± SD. Statistical significance was assessed by one-way ANOVA and Tukey post hoc for comparison within groups. (*, *p* < 0.05, **, *p* < 0.01, ****, *p* < 0.0001)

Previous studies demonstrated that GR activation has anti-migratory effects [39]. Conversely, West and colleagues recently reported that GR induces the expression of genes that are essential for migration and invasion [12, 40]. Similar to other steroid hormone receptors, signaling context and the hormonal milieu (i.e., time and concentration of exposure to either agonist or antagonist) may dictate distinct receptor actions in the same biological system [41]. We tested whether GR differentially responds to acute and chronic effects of Dex by exposing GR+ MDA-MB-231 TNBC cells to a range of pharmacologically relevant Dex concentrations at two different time points: 15 min (“acute”) and 6 h (“chronic”) (Fig. 1a). Our data demonstrate that when cells are pretreated with Dex in charcoal-stripped serum-containing media (i.e., steroid hormone free, but growth factor rich) for 15 min and then subjected to scratch wound assays, subsequent Dex-induced migration is inhibited (Fig. 1b). Conversely, when the same cells are instead pretreated with Dex for 6 h in charcoal-stripped serum-containing media, subsequent treatment with Dex stimulates migration (Fig. 1c). A biphasic response of acute vs. chronic Dex treatment was also observed in Hs578T cells (Supplementary Fig. 1A-C).

To explore signaling pathways that may be altered upon chronic (i.e., 6 h) Dex treatment in TNBC cells, we probed existing microarray data from the GSE113571, in which MDA-MB-231 cells were treated with 100 nM Dex for 4 h [12]. Ingenuity Pathway Analysis was used to determine which pathways were significantly upregulated or downregulated by liganded GR. Notably, the TGFβ1 pathway was among the most significantly upregulated (Fig. 1d). We thus evaluated the requirement for GR in TGFβ1-induced MDA-MB-231 cell migration. Cells were pretreated for 18 h with vehicle, RU486 (i.e., a GR antagonist) alone, Dex alone, TGFβ1 alone, or either agent (Dex or TGFβ1) in combination with RU486. Cells were then subjected to scratch wound assays in the presence of the respective treatments. As expected, we observed pro-migratory effects on cells that were chronically treated with 1 μM Dex (18 h). Cells that were treated for 18 h with 10 ng/mL of TGFβ1 were also more migratory relative to vehicle controls. Surprisingly, RU486 abrogated the pro-migratory effects of either Dex or TGFβ1 (Fig. 1e). Similar results were observed in Hs578T TNBC cells (Supplementary Fig. 1D). Our results suggest that TGFβ1-induced TNBC cell migration is mediated by GR. Notably, TGFβ1-induced cell migration occurred in steroid hormone-free conditions in the absence of exogenously added GR ligands.

### TGFβ1 induces TNBC cell migration via phosphorylation of GR Ser134

**Fig. 2:**
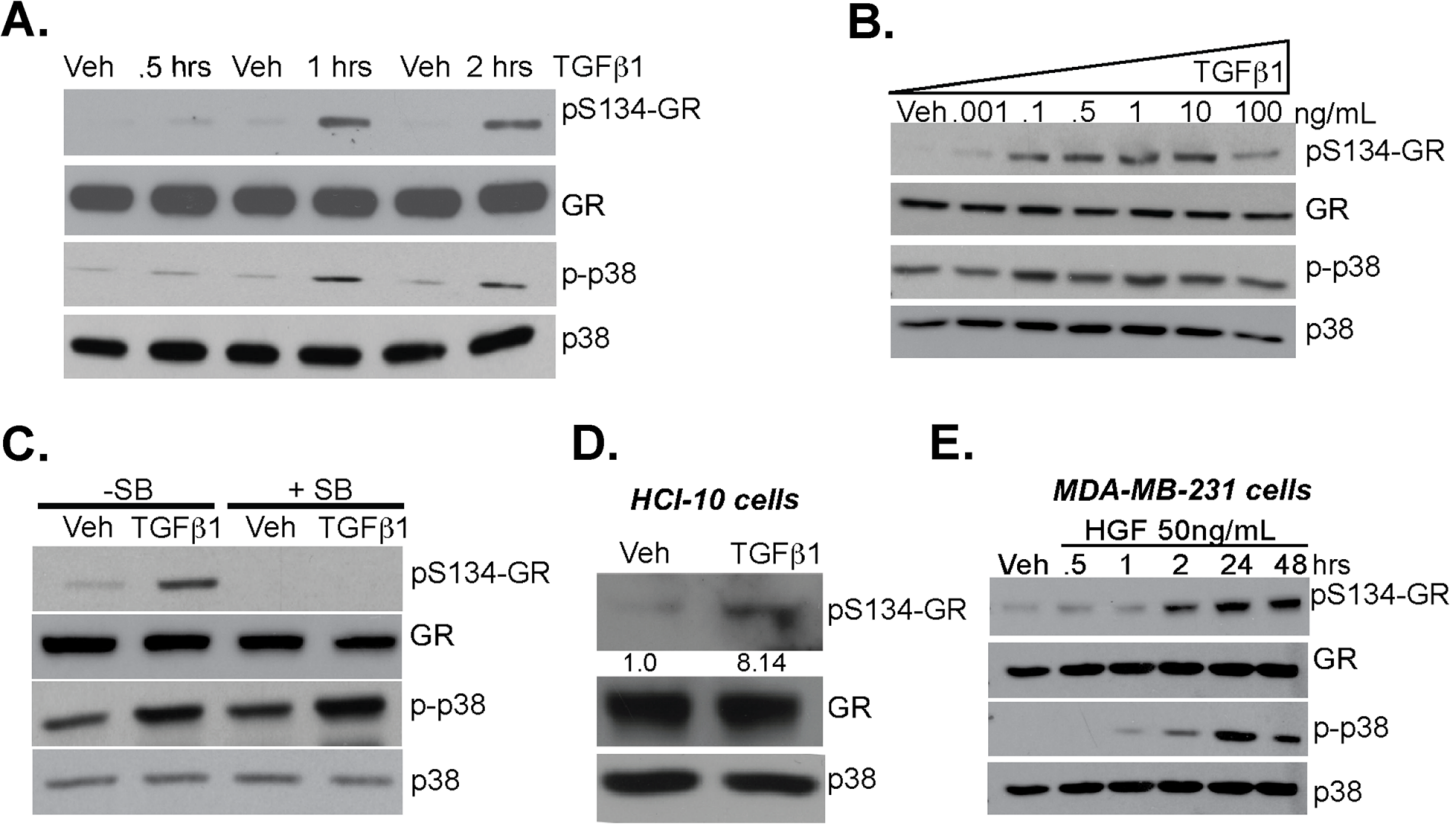
TGFβ1 induces p38 MAPK-dependent phosphorylation of GR Ser134. **a** Representative Western blot analysis of phosphorylated GR (pS134-GR), total GR, phosphorylated p38 MAPK (p-p38 MAPK), and total p38 MAPK protein levels in MDA-MB-231 cells treated with either vehicle control or 10 ng/mL TGFβ1 for 0.5, 1, or 2 h. Representative images of at least three independent experiments is shown (Densitometry of aggregated data from multiple experiments is shown in Supplemental Fig. 2); total p38 serves as a loading control. **b** MDA-MB-231 cells were treated with increasing concentrations of TGFβ1 for 1 h. Western blot analysis shows pS134-GR, total GR, p-p38, and total p38 protein levels (Densitometry of aggregated data from multiple experiments is shown in Supplementary Fig. 2). **c** Representative Western blot analysis of pS134-GR, total GR, p-p38, and total p38 in MDA-MB-231 cells pretreated with either 10 μM SB203580 (p38 inhibitor) or DMSO control for 30 min followed by either vehicle control or 10 ng/mL of TGFβ1 for 1 h. Representative images of at least two independent repeats are shown (Densitometry of aggregated data from multiple experiments is shown in Supplementary Fig. 2); total p38 serves as a loading control. **d** Patient-derive xenograft (PDX) HCI-10 cell line and were treated with 10 ng/mL of TGFβ1 and evaluated for expression of pS134-GR, total GR and total p38 (loading control) by Western blotting. Densitometry for pS134-GR levels are shown relative to vehicle control. **e** Western blot analysis of pS134-GR, total GR, p-p38 MAPK, and total p38 MAPK protein levels in MDA-MB-231 cells treated with either vehicle control or HGF (50 ng/mL) for 0.5, 1, 2, 24, and 48 h. Equal amounts of protein were loaded (Densitometry of aggregated data from multiple experiments is shown in Supplementary Fig. 2); total p38 serves as a loading control

Previously, we reported that cellular stress (i.e., H_2_O_2,_ hypoxia, nutrient starvation) and stressful growth conditions such as growth in low attachment/suspension and chemotherapeutic agents (i.e., Taxol) increase phosphorylation of GR on Ser134, leading to the increased ligand-induced expression of selected GR target genes (e.g., BRK, HIF2, AhR) that are essential for advanced cancer phenotypes in TNBC [16, 17]. Additionally, we reported that pS134-GR species are elevated in TNBC relative to luminal breast cancer subtypes [16]. Since TGFβ1 expression is also elevated in TNBC and associated with poor outcome, we predicted tight linkage between TGFβ1 signaling and phosphorylation of GR Ser134 [42, 43]. MDA-MB-231 cells were treated with 10 ng/mL TGFβ1 for 30 min, 1 h, and 2 h; phosphorylation of GR Ser134 was induced by TGFβ1 at 1 h and 2 h. Additionally, p38 MAPK activity, as measured by active-site phosphorylation, was also elevated at these timepoints (Fig. 2a and see densitometric analyses of aggregated data from multiple repeats in Supplementary Fig. 2a). Dose-response curves demonstrated robust GR Ser134 phosphorylation over a wide range of TGFβ1 doses (0.1–10 ng/ml) (Fig. 2b and see densitometric analyses of aggregated data from multiple repeats in Supplementary Fig. 2B). We then tested the requirement for p38 MAPK in GR Ser134 phosphorylation in TNBC models; MDA-MB-231 cells were pretreated with either vehicle control (DMSO) or SB203580 (p38 inhibitor) for 30 min prior to TGFβ1 exposure for 1 h. While basal levels of p38 MAPK activity remained somewhat elevated, inhibition of p38 MAPK completely blocked TGFβ1 dependent phosphorylation of GR Ser134 (Fig. 2c and see densitometric analyses of aggregated data from multiple repeats in Supplementary Fig. 2C). The specificity of GR Ser134 phosphorylation by p38 MAPK was further explored using SB203580 and SB202190 (p38 inhibitors), LY294002 (Akt inhibitor), or UO-126 (MEK1/2 inhibitor). MDA-MB-231 and Hs578T TNBC cells were pretreated for 30 min with each kinase inhibitor prior to TGFβ1 treatment; TGFβ1-induced GR phosphorylation was exclusively blocked upon inhibition of p38 MAPK, while inhibition of either AKT or ERK1/2 was without effect (Supplementary Fig. 2D and 2E). Similar to MDA-MB-231 and Hs578T cells, TGFβ1 induced robust phosphorylation of GR Ser134 in a TNBC Patient-Derived Xenograft (PDX) model (HCI-10) (Fig. 2d).

In addition to TGFβ1, Hepatocyte Growth Factor (HGF) is a TME factor known to promote cell migration and metastasis [44]. HGF has also been shown to activate p38 MAPK [45]. Given the requirement of p38 MAPK for TNBC motility, we tested the ability of HGF to induce regulated GR phosphorylation [46]. Thus, MDA-MB-231 cells were treated with 50 ng/mL HGF for 0.5–48 h. Robust and persistent phosphorylation of GR Ser134 was observed after 1–2 h (Fig. 2e and Supplementary Fig. 2F). These findings suggest that the TME induces ligand-independent GR Ser134 phosphorylation, nominating GR as an important sensor not only of host/life stress in the form of elevated corticosteroid levels, but for both cellular and local (i.e., TME-derived) cytokine-mediated stress signaling in TNBC cells.

**Fig. 3:**
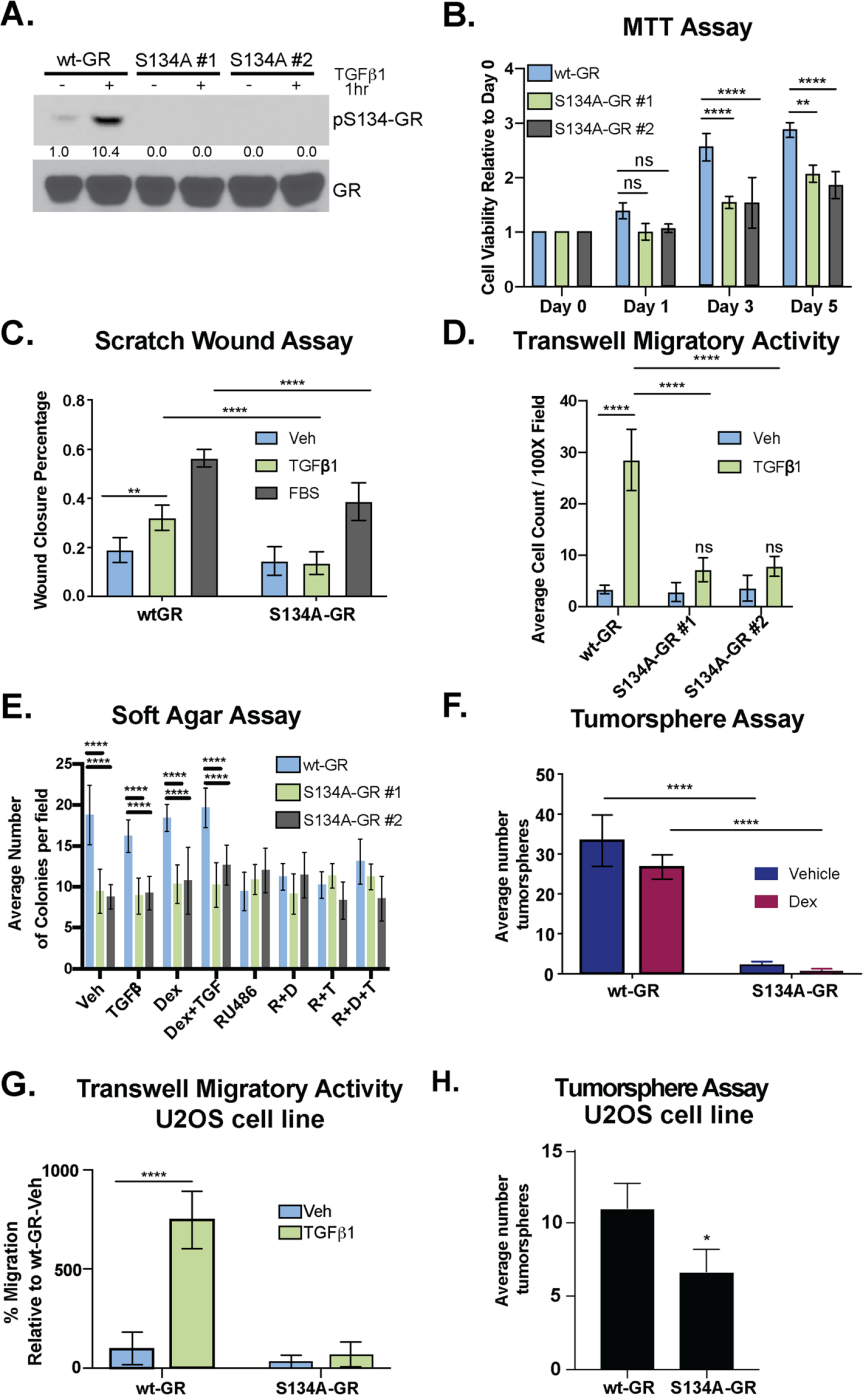
GR Ser134 is required for TGFβ1-mediated migration of TNBC cells. **a** Phosphorylation of GR at Ser134 by 10 ng/mL of TGFβ1 in wt-GR and S134A-GR MDA-MB-231 CRISPR models was examined by Western blot. Densitometry values of the pS134-GR levels are indicated relative to vehicle control. **b** Proliferation of MDA-MB-231 cells expressing either wt-GR or S134A-GR was examined using MTT growth assays. The mean of three biological replicates ± SD is shown. **c** MDA-MB-231 cells expressing either wt-GR or S134A-GR clone #1 were treated with Veh, 10 ng/mL TGFβ1, or 10% FBS overnight and then analyzed by scratch wound assay. Cells were exposed to respective treatments during the course of the migration assay to stimulate migration. The fraction of wound area closure was determined by tracing images in ImageJ. The mean of three field images from each of the three biological replicates is shown ± SD. Significance was assessed by two-way ANOVA and Tukey post hoc for comparison within groups (**, *p* < 0.01 and ****, *p* < 0.0001). **d** Transwell migration assays were used to test the migratory activity of wt-GR and S134A-GR (clone #1 and clone #2) MDA-MB-231 cells with either vehicle or TGFβ1 (10 ng/mL) as the chemoattractant in the bottom chamber. Cells were allowed to migrate for 18 h. The mean of three biological replicates is shown ± SD. Significance was assessed by two-way ANOVA and Tukey post hoc for comparison within groups (****, *p* < 0.0001). **e** Soft-agar colony formation assays were used to test the effect of TGFβ1, Dex, RU486, and their respective combinatorial treatment on MDA-MB-231 cells expressing either wt-GR or S134A-GR (clone #1 and clone #2). The mean of four field images from each of the three biological replicates is shown ± SD. Significance was assessed by two-way ANOVA and Tukey post hoc for comparison within groups relative to Veh wt-GR cells (****, *p* < 0.0001). **f** Secondary tumorsphere of wt-GR or S134A-GR MDA-MB-231 cells. Cells were treated with either Vehicle or 1 μM Dex. Data are presented as the average ± SD of three biological replicates. Significance was assessed by two-way ANOVA and Tukey post hoc for comparison within groups (****, *p* < 0.0001). **g** Transwell migration assays were used to test the migratory activity of U2OS cells expressing either wt-GR or S134A-GR with either vehicle or 10 ng/mL TGFβ as the chemoattractant. The mean of the percentage of three biological replicates is shown ± SD. Significance was assessed by two-way ANOVA and Tukey post hoc for comparison within groups (****, *p* < 0.0001). **h** Secondary tumorsphere of wt-GR or S134A-GR U2OS cells. No treatment was added. Data are presented as the average ± SD of three biological replicates. Statistical significance was assessed by unpaired Student’s *t* test (*, *p* < 0.05)

To test the requirement for GR Ser134 in TGFβ1-regulated TNBC cell migration, we employed a CRISPR/Cas9 approach to create MDA-MB-231 cells expressing either wt-GR (control) or a point mutant GR in which Ser134 has been changed to Alanine (S134A-GR clone #1 and clone #2). We confirmed expression of either endogenous WT or S134A GR via western blotting following treatment of cells with 10 ng/mL of TGFβ1. Predictably, we observed equal levels of total GR in all cell lines, but no signal representing GR Ser134 phosphorylation in TNBC cells harboring S134A-GR (Fig. 3a). Using MTT assays as a readout of cell viability and proliferation, we compared wt-GR and S134A-GR cell lines; cells harboring S134A GR remained viable and proliferated at similar rates relative to wt-GR+ controls (Day 1). However, CRISPR knock-in of S134A GR resulted in reduced cell numbers evident at days 3–5 relative to wt-GR+ controls (Fig. 3b). The migration of these models in response to 10 ng/mL TGFβ1 (18 h) was then measured using scratch wound assays. Fetal Bovine Serum (FBS) (10%) was included as a positive control that contains multiple soluble factors capable of stimulating cancer cell migration. Cells harboring either wt-GR or S134A-GR (clone #1) were pretreated with vehicle control, 10 ng/mL TGFβ1, or 10% FBS overnight; cells were then subjected to scratch wounds, re-treated with their respective treatments and allowed to migrate for 18 h. TGFβ1-mediated migration was significantly impaired in cells expressing S134A-GR relative to wt-GR (Fig. 3c). These findings were replicated (clone #1 and clone #2) in transwell migration assays (Fig. 3d). Migration was stimulated with either vehicle or 10 ng/mL TGFβ1 as the chemoattractant (i.e., added to the bottom chamber). Again, robust TGFβ1-induced migratory activity occurred in wt-GR cells cultured in steroid hormone-free media lacking exogenously added GR ligands (i.e., Dex). However, cells harboring S134A-GR (i.e., two independent CRISPR clones) were significantly less migratory (18 h) relative to cells harboring wt-GR (Fig. 3d). Similarly, we tested the invasive ability of wt-GR and S134A-GR clones. Cells were seeded and allowed to invade through Matrigel inserts towards either vehicle or 10 ng/mL TGFβ1 as the chemoattractant. Predictably, the invasive ability of both S134A GR clones was significantly impaired relative to cells expressing wt-GR (Supplementary Fig. 3).

Anchorage-independent growth in soft agar (i.e., an in vitro measure of cell transformation) is a hallmark of cancer cells that is indicative of the ability to grow and survive independently of basement membrane attachment. TNBC cells expressing S134A-GR exhibited impaired soft-agar colony formation relative to cells expressing wt-GR (Fig. 3e). TNBC cells harboring wt-GR, but not S134A-GR (two clones), formed colonies in media containing steroid hormone-free fetal bovine serum (DCC) with either vehicle control or GR agonists (e.g., Dex). However, while Dex or TGFβ1 did not appreciably increase basal soft-agar colony formation over that observed in DCC alone, the GR antagonist, RU486, blocked soft-agar colony formation, demonstrating the GR-dependence of this cancer cell biology in 3D conditions (Fig. 3e). Similar results were observed in tumorsphere assays in which TNBC cells were plated at limiting dilution in ultra-low attachment dishes as an in vitro readout of cancer stem-like cell behavior [47]; while addition of Dex to sphere media was without effect, MDA-MB-231 cells harboring S134A mutant GR failed to form tumorspheres relative to cells expressing endogenous wt-GR (Fig. 3f). Impaired cell migration as measured by chemotaxis in transwell migration assays (Fig. 3g) as well as attenuated tumorsphere formation were also observed using the previously described GR-null/low U2OS osteosarcoma cell line engineered to stably express S134A-GR relative to wt-GR (Fig. 3h) [15]. These data demonstrate that pS134-GR is critical for TGFβ1-mediated cell migration and basal growth in suspension/3D (anchorage-independent growth and tumorsphere formation), even in the absence of exogenously added GR agonists.

### The pS134-GR binding partner, 14-3-3ζ mediates TNBC cell migration

**Fig. 4:**
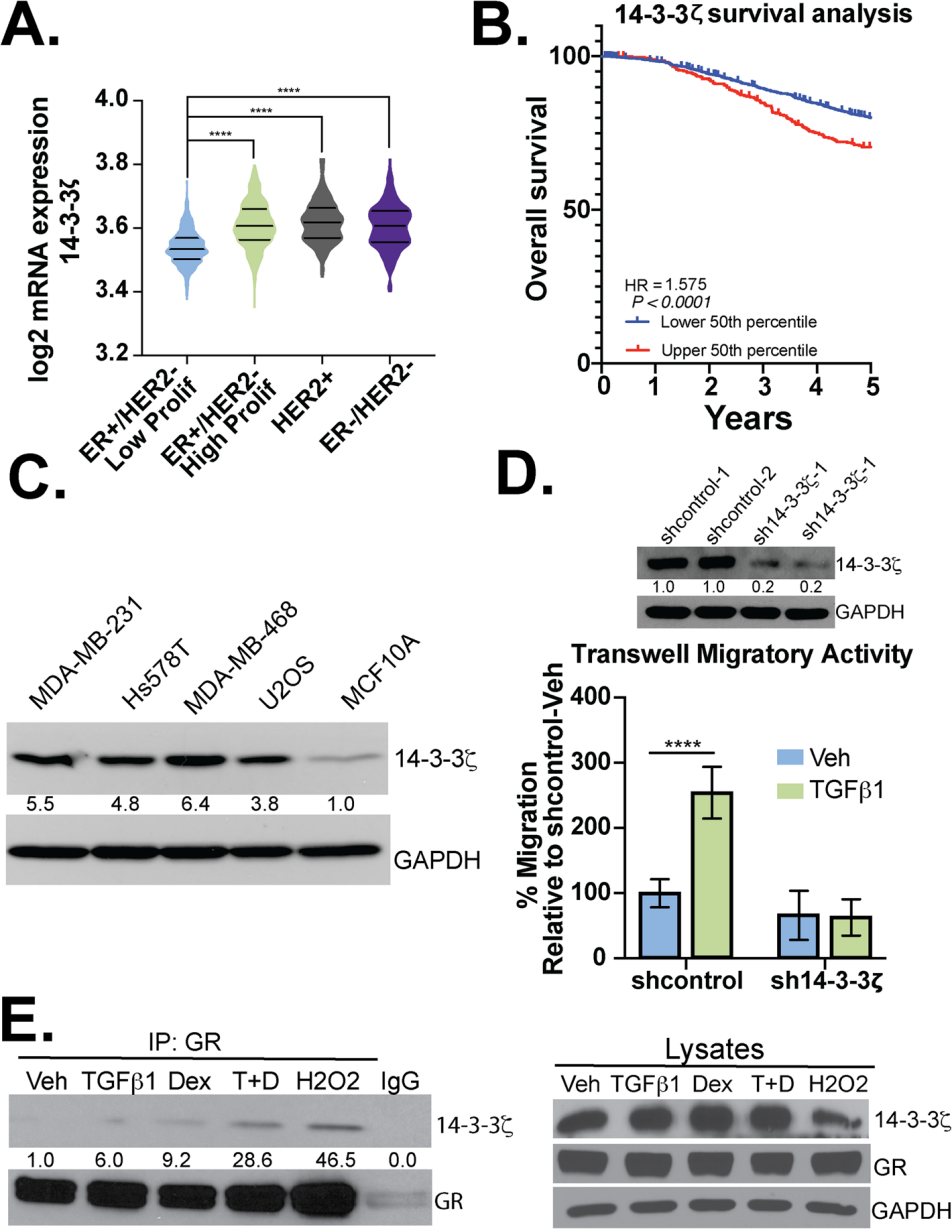
14-3-3ζ is required for TGFβ1-induced TNBC cell migration. **a** Expression of 14-3-3ζ in breast cancer patients was analyzed using the METABRIC database (*n* = 1700). Significance was assessed by one-way ANOVA and Tukey post hoc for comparison between groups (****, *p* < 0.0001). **b** Survival analysis for patients from the METABRIC cohort and with mRNA microarray profiling for 14-3-3ζ were stratified into two groups: expression higher than median mRNA expression of 14-3-3ζ (upper 50th percentile) or lower than median (bottom 50th percentile). The results were analyzed for significance with a logrank *p* value of *p* < 0.0001 (*n* = 1981). **c** Western blot analysis for 14-3-3ζ of different cell lines indicated. Densitometry values for the 14-3-3ζ levels are indicated relative to non-tumorigenic MCF10A cells. **d** Western blot analysis of 14-3-3ζ protein levels in MDA-MB-231 cells expressing either shcontrol or sh-14-3-3ζ; two pools of each respective shcontrol are shown. Densitometry values for the 14-3-3ζ levels are shown relative to shcontrol #1. Transwell migration assays were used to test the migratory activity of shcontrol and sh14-3-3ζ MDA-MB-231 cells. TGFβ1 (10 ng/mL) was used as the chemoattractant and cells were allowed to migrate for 18 h. The mean of three independent biological replicate experiments is shown ± SD. Statistical significance was assessed by two-way ANOVA and Tukey post hoc corrections (****, *p* < 0.0001). **e** Western blot analysis was performed on immunocomplexes and whole-cell lysates to evaluate the interaction of 14-3-3ζ with GR in response to Veh, TGFβ1 (10 ng/mL), Dex (1 μM), TGFβ1+Dex, and H_2_O_2_ (100 μM; positive control for GR phosphorylation). Densitometry values for the 14-3-3ζ levels in the GR IP groups are shown relative to vehicle control. IgG was used as a negative control

14-3-3 proteins are a conserved family of regulatory molecules that bind to diverse signaling proteins in eukaryotic cells. Galliher-Beckley et al., first reported that 14-3-3ζ interacts with GR in a pS134-GR-dependent manner in U2OS osteosarcoma cells [15]. To evaluate the role of 14-3-3ζ in GR+ TNBC, we probed 14-3-3ζ mRNA expression levels across breast cancer subtypes in the TCGA dataset and stratified patients based on the expression levels of ER, PR and HER2 receptors as determined by clinical immunohistochemistry analyses of primary tumors. Data from METABRIC were obtained from cBioPortal. The expression of 14-3-3ζ mRNA in patients with ER−/HER2- (TNBC) or Her2+ breast cancer was higher when compared to patients with ER+/HER2- breast cancer (Fig. 4a). Similarly, high levels of 14-3-3ζ mRNA, as separated by the median cutoff of mRNA expression, correlate with poor overall survival in patients within the METABRIC cohort (Fig. 4b). Accordingly, we observed abundant expression of 14-3-3ζ in several breast cancer cell lines, including MDA-MB-231, Hs578T, and MDA-MB-468 (TNBC) cells as well as U2OS osteosarcoma cells (positive control). Weak 14-3-3ζ expression was observed in non-tumorigenic MCF10A cells (Fig. 4c). To test the requirement for 14-3-3ζ in TGFβ1-mediated migration, we generated MDA-MB-231 cells stably expressing two distinct 14-3-3ζ-targeted shRNAs or a non-targeting vector control (shcontrol); 14-3-3ζ knockdown was confirmed in two separate pools by Western blotting (Fig. 4d; upper panel). TGFβ1 induced robust cell migration in shcontrol but not sh14-3-3ζ TNBC models (Fig. 4d). We confirmed that 14-3-3ζ/GR interaction occurs in TNBC models (Fig. 4e); MDA-MB-231 cells were subjected to treatment with either Veh, TGFβ1, Dex, TGFβ1+Dex, or H_2_O_2_ (a potent inducer of GR Ser134 phosphorylation). Similar to that observed in H_2_O_2_-treated U2OS osteosarcoma cells [15], co-immunoprecipitation assays revealed modest TGFβ1 or Dex-regulated interaction between GR and 14-3-3ζ that was further enhanced following combinatorial treatment of cells with both TGFβ1 and Dex. These data suggest that 14-3-3ζ and pS134-GR function in the same signaling pathway and/or cooperate to induce altered gene expression and subsequent migration of TGFβ1-responsive TNBC cells.

### The GR transcriptome implicates MAPK-driven signaling in TNBC migration

**Fig. 5:**
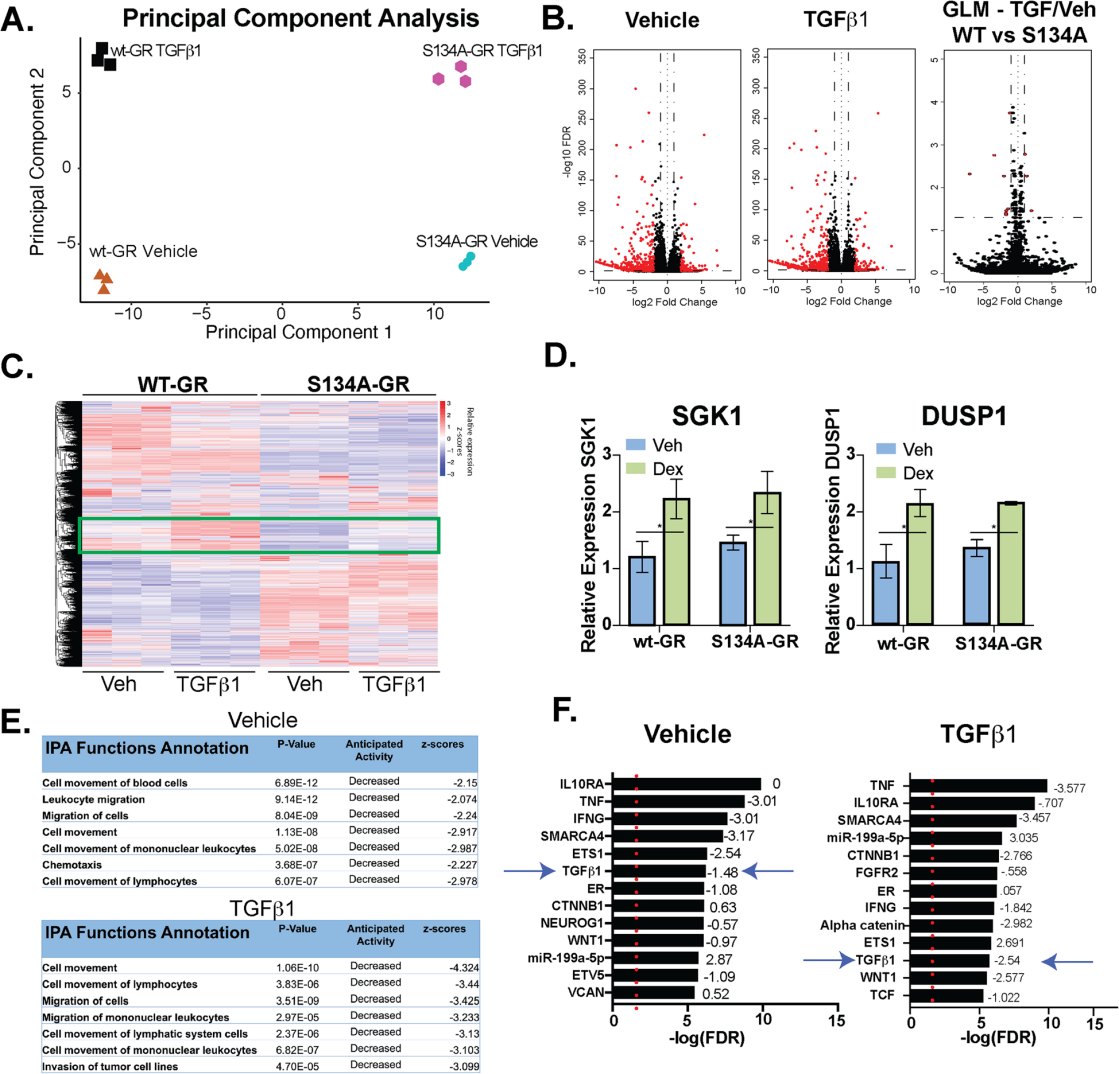
WT vs. S134A-GR transcriptomes in TGFβ1-treated MDA-MB-231 cells. **a** Principal component analysis plot derived from RNA-seq shows the differences in transcriptomes between MDA-MB-231 cells expressing either WT or S134A-GR cells and treated with vehicle or 10 ng/mL TGFβ1 for 6 h. **b** Volcano plot showing differential expression of genes in wt-GR+ compared to S134A-GR+ TNBC cells treated for 6 h with either Vehicle (left) or 10 ng/mL of TGFβ1 (middle). Volcano plot using the generalized linear model approach in EdgeR (comparisons of wt-GR and S134A-GR in response to TGF) to illustrate the effects of TGFβ1 treatment of cells expressing wt-GR relative to cells expressing S134A-GR (right). Red dots indicate genes with an absolute log2 fold-change of 1.0 or more and a false discovery rate of less than 0.05. Genes for GLM are detailed in Supplementary Table 1A. **c** Supervised heatmap shows significant differences in gene expression as measured by RNA-seq in TGFβ1-treated MDA-MB-231 cells expressing either wt-GR or S134A-GR. Rectangle indicates a cluster of interest in which genes are upregulated in response to TGFβ1 in wt-GR cells but not in the S134A-GR cells. All genes that were used for differential expression analysis are represented in this heatmap (*n* = 19,760). **d***DUSP1* and SGK1 mRNA levels were assessed using qRT-PCR following normalization to *18S* expression. Mean expression of three independent replicates ± SD is shown. **e** Cell function and mechanism analyses of pathways via IPA of differentially expressed genes with an absolute log fold-change of 1.5 or more and a *p*-adjusted value of less than 0.05 in wt-GR relative to S134A-GR TNBC cells treated for 6 h with either Vehicle or TGFβ1 (10 ng/mL). **f** Upstream regulator analyses via IPA of differentially expressed genes with an absolute log fold-change of 1.5 or more and a *p*-adjusted value of less than 0.05 in wt-GR relative to S134A-GR TNBC cells treated with either Vehicle or 10 ng/mL of TGFβ1. Activation/inhibition *z*-scores are shown as well. Red dotted line indicates the significance value of 1.3 (*p* < .05)

Our data (Figs. 1, 2, 3, and 4) suggest that significant ligand-independent gene expression occurs via phosphorylation of GR Ser134. To further expand our understanding of the impact of pS134-GR on TNBC cell behavior, we performed RNA-seq studies in MDA-MB-231 cells expressing either wt-GR or S134A-GR (clone #1). Cells were treated with either vehicle, to capture basal homeostatic (i.e., ligand-independent) GR actions or with TGFβ1, as a regulated input to p38 MAPK activation and subsequent GR phosphorylation (6 h). Principal component analysis demonstrated intrinsic differences between both vehicle-treated and TGFβ1-treated models (Fig. 5a). Remarkably, in the absence of a specific stimulus, the basal transcriptome of cells expressing S134A GR differs dramatically from that of cells expressing wt-GR. These data suggest that GR Ser134 acts as a homeostatic “sensor” of numerous cell-intrinsic and cell-extrinsic signaling inputs to p38 MAPK, accounting for significant ligand-independent or “basal” differences between vehicle-treated models. Volcano plots were used to further illustrate the effect of S134A-GR single point mutation in vehicle or TGFβ1-treated cells. Namely, vehicle-treated models clearly exhibited a broad array of transcription-wide differences, while fewer changes in mRNA expression were attributed to TGFβ1 treatment between wt and S134A GR+ models, as measured using the generalized linear model framework (GLM) in EdgeR (Fig. 5b). GLM analysis identified a limited number of differentially expressed genes in response to TGFβ1 when comparing wt-GR+ and S134A-GR+ models. Notably, *LEFTY1*, *COL1A1*, and *OLFM2*, known mediators of TGFβ1 signaling, were identified as differentially expressed (red dots) using the GLM approach (Supplementary Table 1A) [48, 49]. Ingenuity Pathway Analysis (IPA) of this limited gene subset identified ERBB2 as well as TGFBR1/TGFβ1 (i.e., both receptor and cognate ligand) signaling as potential S134A GR-regulated pathways (Supplementary Table 1B). Consistent with these results, comparison of the response to TGFβ1 between wt and S134A GR models also revealed a subset of ~ 80 TGFβ1-induced genes in cells harboring wt-GR that were not regulated by TGFβ1 in cells expressing S134A-GR (Fig. 5c green rectangle and Supplementary Fig. 4A). Importantly, ligand-dependent GR activation (i.e., in Dex-treated cells) remained similar across these models as measured by qPCR analysis of two well-characterized GR target gene (DUSP1 and SGK1) mRNAs, indicating that ligand-bound S134A-GR is not transcriptionally impaired (Fig. 5d). Further studies are needed to evaluate the role of Ser134 in the global transcriptional response of liganded GR.

Ingenuity Pathway Analysis and Gene Set Enrichment Analysis (GSEA) tools were then used to evaluate the impact of GR Ser134 modification on ligand-independent basal (Veh) and TGFβ1-induced cellular pathways/behaviors. IPA analysis revealed that cellular migration is strongly represented in the genes that are primarily regulated in cells expressing wt-GR, but not in cells expressing S134A GR (i.e., gene sets associated with cell migration are decreased in S134A-GR TNBC cells regardless of treatment; Fig. 5e and Supplementary Fig. 4B). This finding further strengthens our hypothesis that phosphorylation of GR on Ser134 is critical for migration of TNBC cells. Interestingly, the TGFβ1 pathway itself is significantly downregulated in S134A-GR cells, and consistent with these results, this is also readily apparent in TGFβ1-treated cells, as was independently identified via both the upstream regulator analysis in the IPA platform and METACORE (Fig. 5f and Supplementary Fig. 4C). In sum, the transcriptomes of cells harboring either wt-GR or S134A-GR are remarkably different basally, perhaps because GR Ser134 acts as an important ligand-independent sensor of multiple homeostatic intrinsic (basal) and extrinsic (TME-derived factors) cellular stress inputs to the p38 MAPK pathway [15, 16].

**Fig. 6:**
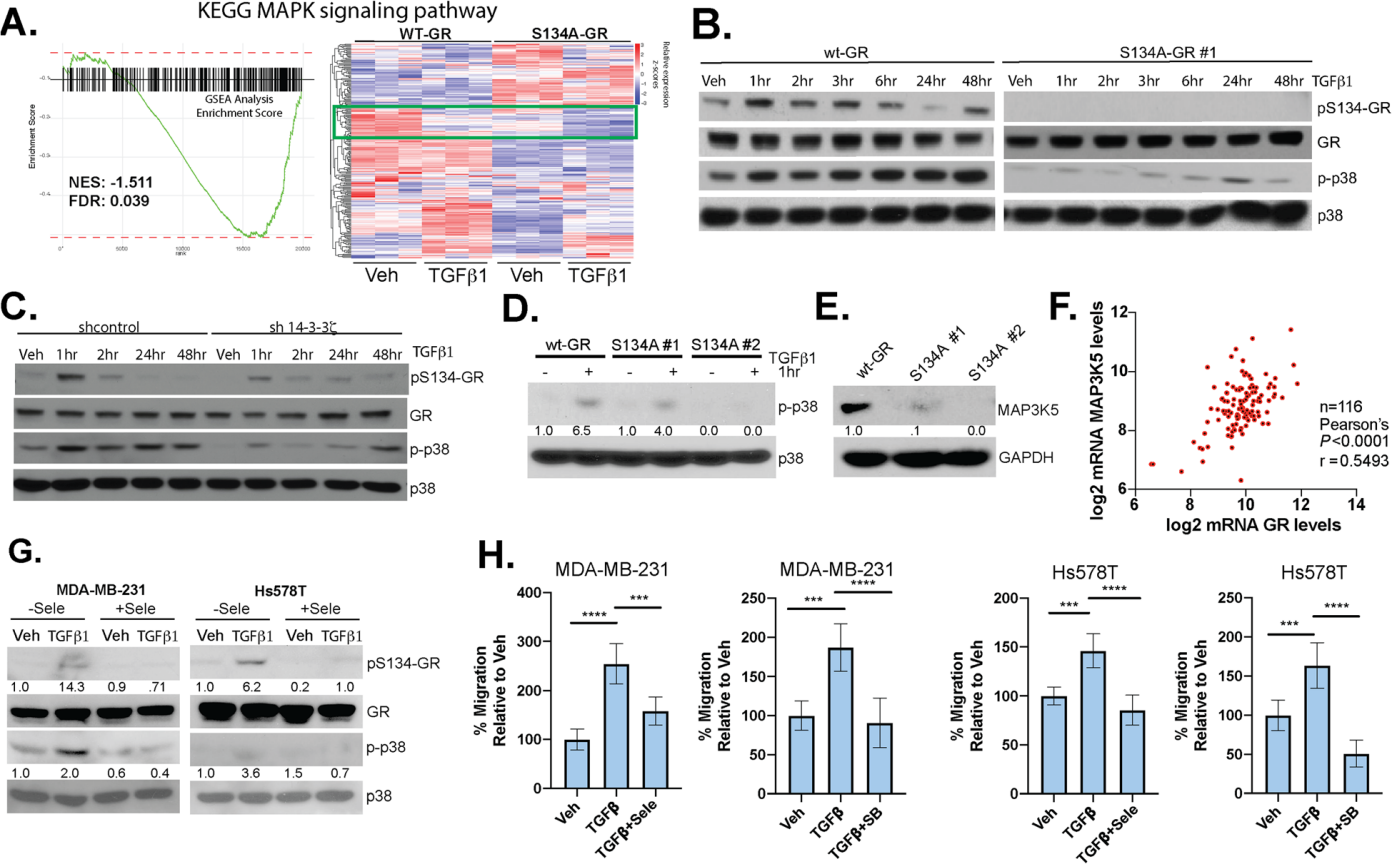
Phosphorylation of GR Ser134 is critical for MAPK signaling. **a** Gene Set Enrichment Analysis and associated MAPK gene heat map reveal a dramatic loss of expression of genes important for MAPK signaling in TNBC cells expressing S134A-GR compared to wt-GR. **b** Representative Western blot analysis of pS134-GR, total GR, p-p38, and total p38 protein levels (loading control) in MDA-MB-231 cells expressing either wt-GR or S134A GR clone #1, and **c** either shcontrol or sh-14-3-3ζ . Cells were treated with 10 ng/mL TGFβ1 at the indicated timepoints. Representative experiments of at least three independent repeats are shown. **d** WT-GR or S134A GR clone #1 and clone #2 were treated with 10 ng/mL TGFβ1 to confirm previous findings with regard to p38 MAPK phosphorylation. Densitometry values for the p-p38 levels are shown relative to vehicle control in wt-GR. **e** MAP3K5 protein expression is shown for wt-GR, S134A clone #1, and S134A clone #2. Densitometry values for the MAP3K5 levels are shown relative to wt-GR. **f** TCGA normalized data was log2 transformed for TNBC patients. Correlation between MAP3K5 and GR was calculated by Pearson’s correlation. **g** Western blot analysis of pS134-GR, total GR, p-p38, and total p38 in MDA-MB-231 cells pretreated with either 10 μM selonsertib (MAP3K5) or DMSO control for 30 min followed by either vehicle control or 10 ng/mL of TGFβ1 for 1 h. Total p38 serves as a loading control. Densitometry values for pS134-GR and p-p38 levels are indicated relative to vehicle control of each cell line. **h** Transwell migration assays were used to test the migratory activity of MDA-MB-231 cells in response using either vehicle or TGFβ1 (10 ng/mL) as the chemoattractant in the bottom chamber. Additionally, cells were treated with either 10 μM SB203580 (SB) or 10 μM selonsertib (Sele) in both the upper and lower chambers. Cells were allowed to migrate for 18 h. The mean of the percentage of three biological replicates is shown ± SD. Significance was assessed by Two-way ANOVA and Tukey post hoc for comparison within groups (***, *p* < 0.001,****, *p* < 0.0001)

Using GSEA analysis for the Kyoto Encyclopedia of Genes and Genomes (KEGG) pathways study, we identified the MAPK pathway as significantly downregulated in cells harboring S134A-GR (Fig. 6a and Supplementary Table 2). Interestingly, we found that there are also basal differences in the MAPK KEGG module (Fig. 6a). This indicates that one or more key components of the MAPK pathway are likely to be regulated by GR Ser134 phosphorylation. Indeed, TGFβ1-induced activation of p38 MAPK, as measured by via active-site phosphorylation, appears to be arrested in cells expressing S134A GR relative to cells expressing wt-GR (Fig. 6b and see densitometric analyses of aggregated data from multiple repeats in Supplementary Fig. 5A). Similarly, 14-3-3ζ knockdown resulted in greatly decreased TGFβ1-induced p38 activation with no change in total p38 expression levels (Fig. 6c and see densitometric analyses of aggregated data from multiple repeats in Supplementary Fig. 5B). Consequently, sh14-3-3ζ cells also exhibited less pS134-GR. To confirm these findings, we tested for phosphorylation of p38 MAPK in response to TGFβ1 and found that it is greatly attenuated in both S134A GR+ clones (Fig. 6d). These data suggest that pS134-GR is an important regulator of one or more kinases upstream of p38 MAPK (i.e., that are required for p38 activation). We thus probed our RNA-seq data within the MAPK pathway KEGG signature, thereby nominating the mitogen-activated protein kinase kinase kinase 5 (MAP3K5, also called MEKK5 or ASK1) as a possible factor that integrates these pathways. MAP3K5 interacts with 14-3-3ζ to facilitate activation of downstream kinases MEK3/6 and p38 MAPK [50]. Indeed, MAPK3K5 protein levels were significantly downregulated in two clones harboring S134A-GR relative to wt-GR (Fig. 6e). Moreover, in an independent experiment using S134A-GR clone #1, we observed downregulation of MAP3K5 at both the mRNA and protein levels (Supplementary Fig. 5C). Although known to be associated with other cancer types, to our knowledge, MAP3K5 has not been implicated in breast cancer. Similar to expression of pS134-GR and 14-3-3ζ (above), MAP3K5 mRNA levels are elevated in TNBC relative to other breast cancer subtypes represented in the METABRIC (Supplementary Fig. 5D), and MAP3K5 and GR mRNA expression levels are strongly correlated in TNBC patients (Fig. 6f) [16]. Notably, inhibition of MAP3K5 with the selective inhibitor, selonsertib, blocked phosphorylation of GR (Fig. 6g) and inhibited TGFβ1-induced migration (Fig. 6h) in both MDA-MB-231 and Hs578T cells. Similarly, inhibition of p38 MAPK using SB203580, halted TGFβ1-mediated migration in both TNBC models (Fig. 6h). Taken together, these results indicate that phosphorylation of GR on Ser134 is critical for the expression of key MAPK pathway intermediates. Specificity controls indicated that serum-induced activation of JNK and p42/p44 MAPKs (ERK1/2) were unaffected in TNBC cells harboring S134A GR (Supplementary Fig. 5E), suggesting that pS134-GR is a highly selective regulator of the p38 MAPK “module” characterized by the three kinase cascade initiated by MAP3K5 in TNBC cells. While the TGFβ1-induced activation of SMADs is thought to occur independently of p38 MAPK, we observed a slight attenuation of TGFβ1-induced SMAD2 phosphorylation (i.e., primarily apparent after 2 h) in cells harboring S134A-GR (Supplementary Fig. 5F). These data suggest that pS134-GR may potentiate sustained SMAD2 phosphorylation and activation, perhaps in part via regulation of the signaling components required for intact p38 MAPK signaling (Fig. 6). We were unable to detect a physical interaction between GR and SMADs (not shown); further studies are needed to address weather GR crosstalk with the TGFβ1 signaling occurs at the level of SMAD regulation.

### A pS134-GR gene signature predicts poor survival in breast cancer patients

**Fig. 7:**
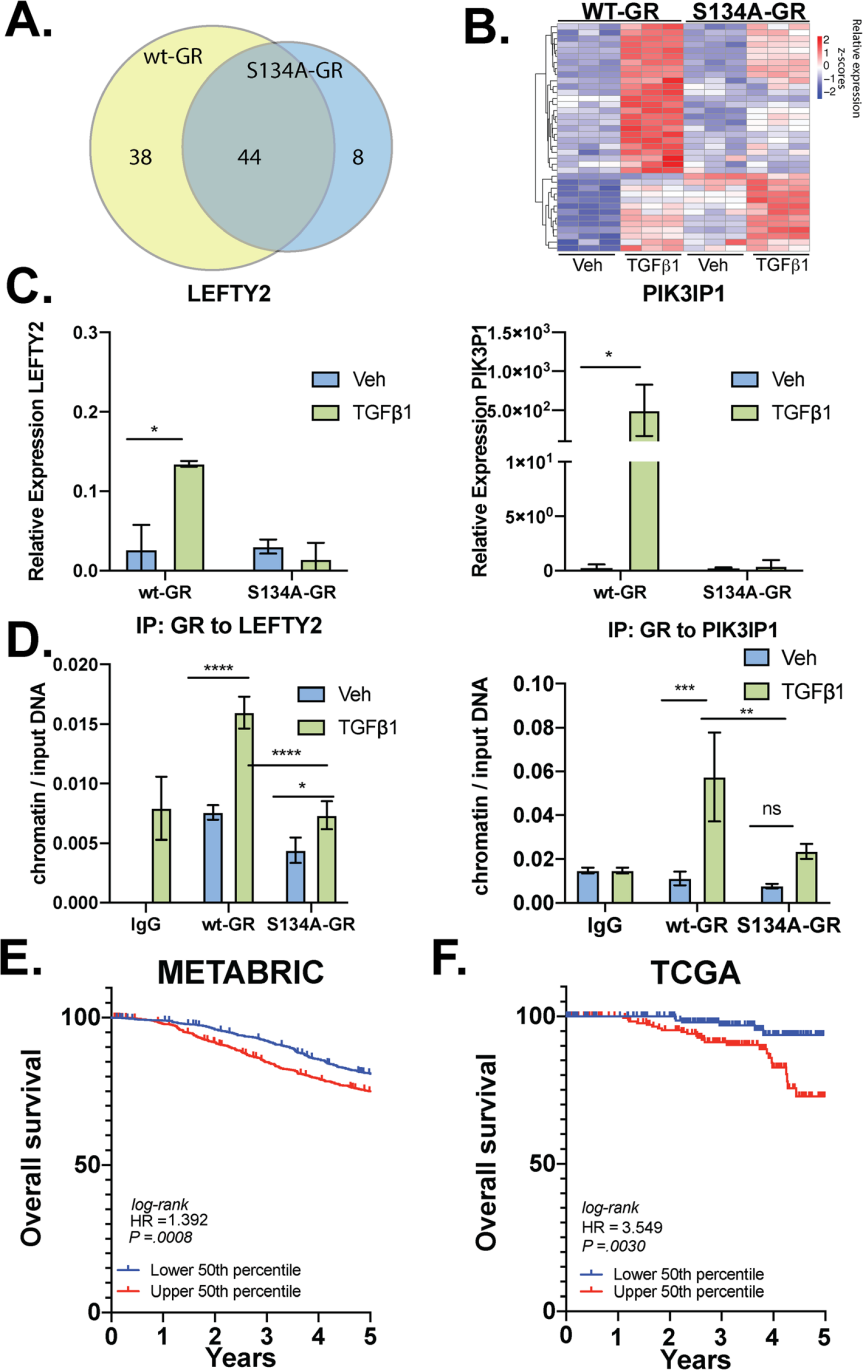
P-S134-GR promotes the expression of a 24-gene signature that correlated with poor prognosis in BC. **a** Venn diagram showing genes that are upregulated by TGFβ1 in MDA-MB-231 cells expressing either wt-GR or S134A-GR. The cutoffs used to define upregulation were a Benjamini-Hochberg value of less than 0.05 and a log2 fold-change of at least 1.5. **b** Supervised clustering of the 39 genes significantly upregulated in wt-GR cells by TGFβ1. **c***LEFTY2* and *PIK3IP1* mRNA levels were assessed by qRT-PCR following normalization to *Actin.* Wt-GR and S134A-GR MDA-MB-231 cells were treated with either vehicle or TGFβ1 (10 ng/mL) for 6 h. Mean expression of three independent replicates ± SD is shown. Statistical significance was assessed by two-way ANOVA and Tukey post hoc for comparison within groups (*, *p* < 0.05). **d** Either wt-GR or S134A-GR MDA-MB-231 cells were treated with either vehicle or TGFβ1 (10 ng/mL) for 1 h. ChIP assays for the LEFTY2 and PIK3IP1 promoters to evaluate recruitment of GR were performed. The mean of three independent biological replicates are shown ± SD. Statistical significance was assessed by two-way ANOVA and Tukey post hoc for comparison within groups (*, *p* < 0.05; ****, *p* < 0.0001). **e** Kaplan-Meier curves are shown for the METABRIC dataset (*n* = 1904). Patients were separated by calculating the median expression of the previously identified GR Ser134-dependent 24-gene signature. This analysis was limited to 5 years of survival data. The results are significant with a logrank *p* value of *p* = .0008. **f** SurvExpress [51] was used to stratify TCGA breast cancer cohort based on their median prognostic index as determined by SurvExpress with the TGFβ1 pS134-GR 24-gene signature. The results were analyzed for significance with a logrank *p* value of *p* = .0030

We speculated that pS134-GR-regulated gene sets important for TGFβ1 signaling may predict prognosis and disease progression in patients with breast cancer. Using our transcriptome (RNA-seq) data, we further evaluated all genes that were uniquely upregulated in cells expressing wt-GR but not S134A GR (i.e., those genes whose TGFβ1-induced regulation requires an intact GR Ser134). Thus, we compared the expression of genes between the vehicle and TGFβ1-treated groups for each TNBC cell line. Using a cutoff of log2 fold-change of + 1.5 and a Benjamini-Hochberg *p* value of 0.05, we identified all TGFβ1-upregulated genes relative to vehicle controls. A total of 90 genes were upregulated by TGFβ1 in MDA-MB-231 cells expressing either wt-GR or S134A GR. Of these TGFβ1-induced genes, 38 were upregulated only in cells harboring wt-GR, and 8 were significantly upregulated only in cells expressing S134A-GR, while 44 genes were upregulated in both cell lines (Fig. 7a). A heatmap approach was used to plot the log2 normalized expression values of the 38 genes upregulated by TGFβ1 in cells expressing wt-GR but not S134A-GR (Fig. 7b and Supplementary Table 4). We further identified a 24-gene cluster of TGFβ1-regulated genes that require GR Ser134 using the same threshold of log2 fold-change of > 1.5 and a Benjamini-Hochberg *p* value of 0.05 (Supplementary Table 3 and 4). Representative genes (*LEFTY2*, *PIK3IP1*) were further validated by qPCR in TGFβ1-treated (10 ng/mL; 6 h) cells. As predicted by our RNA-seq data, we observed TGFβ1-induced upregulation of both *LEFTY2* (~ 4.3 fold increase) and *PIK3IP1* (~ 200 fold increase) in cells expressing wt-GR, but not in cells expressing S134A-GR (Fig. 7c). Additionally, using ChIP assays, we demonstrated robust recruitment of wt-GR species to glucocorticoid response element (GRE)-containing promoter regions of both *LEFTY2* and *PIK3IP1* in response to TGFβ1 alone. However, consistent with our qPCR results, recruitment of S134A GR to these regions was significantly diminished (*LEFTY2*) or failed to occur (*PIK3IP1*) in CRISPR models expressing phospho-mutant GR (Fig. 7d). These data indicate that in the absence of ligand, pS134-GR is recruited to both *LEFTY2* and *PIK3IP1* in response to TGFβ1. Further studies are needed to evaluate the impact of pS134-GR on the global cistrome in the context of TNBC.

To further evaluate the importance of the pS134-GR gene signature in breast cancer patients, we used the METABRIC dataset to calculate the average expression of the above defined 24 pS134-GR-induced genes for each patient tumor and stratified patient populations based on a median cutoff for the average expression of the gene signature. Using Kaplan-Meier curves for analyses of overall survival, we observed that patients whose breast tumors exhibited an average expression of the gene signature within the upper 50th expression percentile experienced poor survival relative to patients whose tumors fell within the lower 50th percentile (Fig. 7e). This difference was significant with a log rank *p* value of 0.0008 (Fig. 7e). To confirm our findings in the METABRIC dataset, we employed the SurvExpress tool with the TCGA breast cancer dataset [51] and observed similar results with significant separation based on overall survival (Fig. 7f). These results suggest that the presence of pS134-GR, as measured using a 24-gene signature, confers poor prognosis in breast cancer patients, regardless of subtype. More studies are needed to further explore the utility of tracking either pS143-GR or pS134-GR gene signatures as potential biomarkers of advanced cancer behaviors (i.e., p38 MAPK-mediated tumor cell dissemination) in breast cancer patient subpopulations.

## Discussion

Herein, we report that TGFβ1 promotes p38 MAPK-dependent phosphorylation of GR Ser134 in TNBC models. Our studies demonstrate that ligand-independent but p38-driven activation of pS134-GR represents a convergence of stress-activated signaling pathways with cellular homeostatic responses, including to local pro-inflammatory cytokines and growth factors (Fig. 2 and Supplementary Fig. 2). We conclude that pS134-GR is a critical effector of migratory and invasive TNBC cell behaviors linked to TGFβ1 signaling in TNBC models, in part explaining why high GR expression predicts poor outcome in women diagnosed with TNBC [7]. Our results suggest that pS134-GR promotes basal TNBC cell survival and TGFβ1-induced migration/invasion even in the absence of exogenously added GR ligands, but rather in response to intrinsic or TME stressors; a pS134-GR gene signature distinct from that induced by Dex [12] predicts poor outcome across all breast cancer subtypes. Taken together, our data suggest that targeting pS134-GR and associated p38 MAPK signaling pathway effectors (MAP3K5) downstream of TGFβ1 or related factors in TNBC patients could be highly beneficial.

Breast cancer patients are frequently treated with Dex (i.e., a potent GR agonist) to alleviate common side effects of chemotherapy. However, in the context of cancer, Dex can promote migration and importantly, pS134-GR (activated-GR) dramatically alters basal and cytokine-induced gene expression in the absence of abundant GR ligands (Fig. 5). Namely, pS134-GR (but not Dex) is required for TNBC cell migration (Fig. 3c and Fig. 3d), invasion (Supplementary Fig. 3), anchorage-independent growth (Fig. 3e), and tumorsphere formation (Fig. 3f), in vitro readouts of advanced cancer behaviors that track with metastatic potential [22]. Our findings are clinically relevant since GR appears to have an active role in the progression of TNBC [12]. Our data suggest that TNBC patients may have high expression of functionally active pS134-GR even when GR agonists are limiting. The presence of elevated pS134-GR levels in TNBC relative to luminal BC cases [16] may explain why GR expression correlates with poor prognosis in TNBC but tracks with good prognosis in luminal BC [7]. Interestingly, GR Ser134 contributes to basal anchorage-independent cell growth (Fig. 3e), a measure of both survival and proliferation. We were surprised that in the presence of TGFβ1 (or other cell-intrinsic or cell-extrinsic agents that activate p38), exogenously added Dex was not required/dispensable for pS134-GR-driven gene expression and TNBC cell migration/invasion in vitro. Further studies are needed to test if TNBC cells (in vitro) or tumor/stromal tissues (in vivo) produce measurable GR ligands and if so, at what intracellular or intratumoral concentrations. Additionally, although our data showed that both wt-GR and S134A-GR respond similarly to Dex at well-characterized GR target genes (*SGK1* and *DUSP1*), additional Dex-regulated genes are likely to be highly sensitive to modification of GR Ser134 [15]. Further studies are needed to address this knowledge gap.

**Fig. 8:**
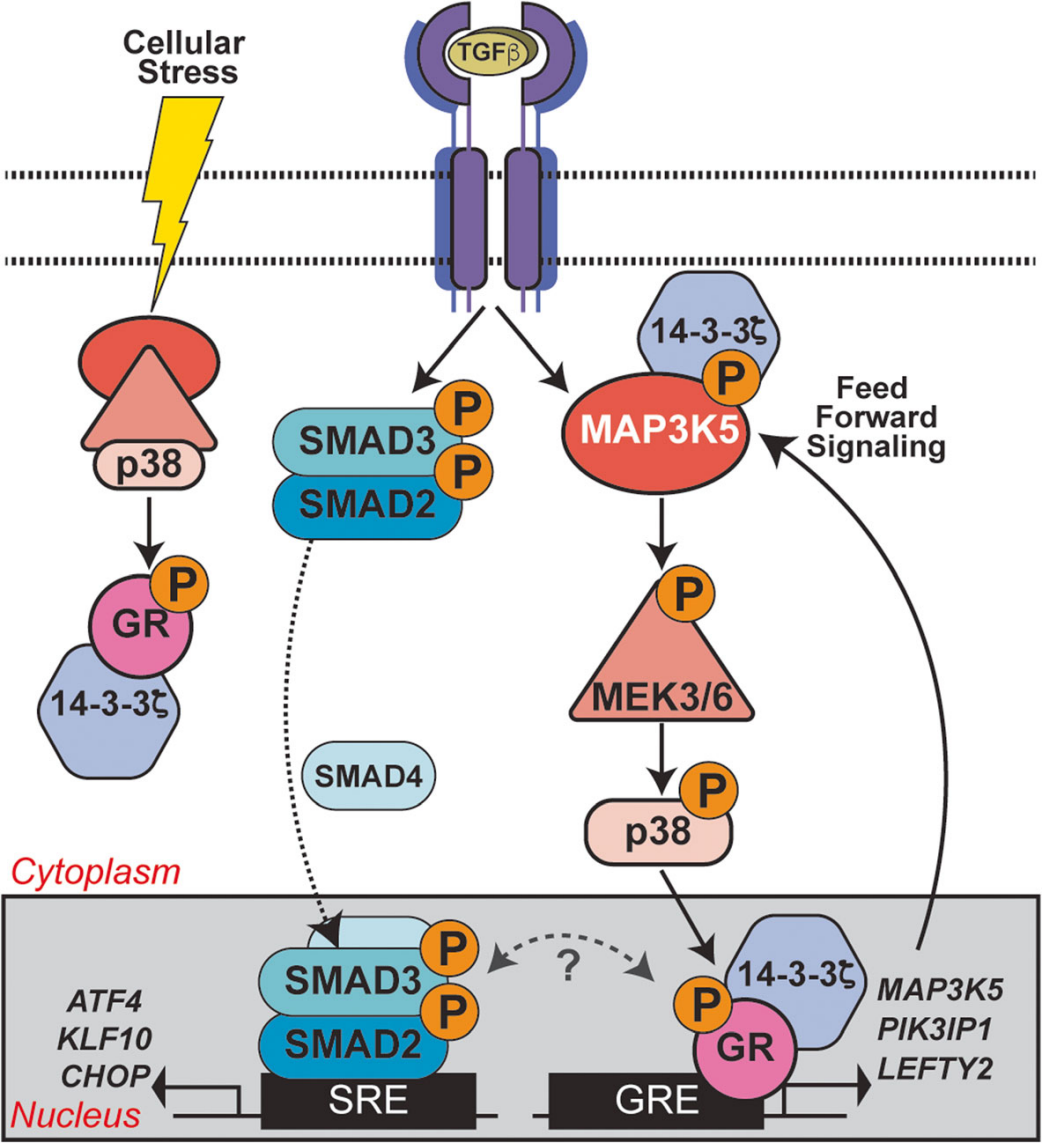
GR Ser134 phosphorylation creates a feedforward signaling loop that potentiates further activation of the p38 MAPK pathway downstream of TGFβ1 in TNBC models**.** Schematic of the conserved three kinase cascade that represents the p38 MAPK module (i.e., MAP3K5, MEK3/6, and p38 MAPK) and known cooperation between 14-3-3ζ and MAP3K5 [50]. Cellular stress as well as TME-derived factors (TGFβ1) input to activation of SMADs and p38 MAPK signaling, resulting in phosphorylation of GR on Ser134. Ligand-independent pS134-GR target genes include key components of the p38 MAPK pathway (MAP3K5) needed for intact p38 signaling. Potential cooperation of 14-3-3ζ and pS134-GR with the SMAD-dependent arm of the TGFβ1 signaling pathway is shown (dotted two-way arch under question mark).

Posttranslational modifications of SRs expressed in breast cancer cells are predicted to have tremendous impact on the clinical course and tumor characteristics [52]. For example, Ser294-phosphorylated PRs regulate gene expression that modulates luminal breast cancer stem/stem-like cell properties and promotes tumor cell plasticity and therapeutic resistance [14]. GR is closely related to PR within the SR superfamily. GR and PR recognize the same DNA binding sites in chromatin, regulate many of the same genes, and even bind many of the same ligands such as RU486 (mifepristone) with similar affinities [53]. Interestingly, pS134 GR-driven anchorage-independent growth was insensitive to Dex but was blocked by RU486 (Fig. 3e). Similarly, we previously described ligand-independent but MAP kinase-dependent actions of pS294-PR in luminal BC models and tumor tissues [54]. Our RNA-seq studies revealed that intact GR Ser134 is required for the expression of genes that promote MAPK activity in TNBC (Fig. 6). A growing body of literature implicates MAPK signaling in cellular processes required for dangerous breast cancer progression, including EMT, stemness, and resistance to chemotherapy [55]. Importantly, inhibition of p38 MAPK activity has led to decreased migration in breast cancer cell lines [46] including MDA-MB-231 and Hs578T herein (Fig. 6h). Our studies revealed that MAP3K5 expression is regulated by pS134-GR. MAP3K5 is essential for the activation of downstream MEK3/6 (MKK3/6) which in turn activates p38 MAPK in the 3-kinase cascade or module required for phosphorylation of GR Ser134 [50, 56]. Notably, because MAP kinase kinase kinase (MKKK) expression is typically limited (i.e., protein expression is low relative to that of MKKs or MAPKs) [57], a relatively modest change in MKKK (i.e., the top of the 3-kinase cascade; MAP3K5) abundance may confer large changes in activation of MAP kinase (i.e., the bottom of the 3-kinase cascade; p38) (Fig. 8). Overall, our data support the existence of a potent feedforward loop in response to TGFβ1 and other TME-derived agents that activate p38 MAPK; p38 MAPK-dependent GR Ser134 phosphorylation promotes increased expression of MAP3K5, thereby reinforcing robust MEK3/6 and p38 MAPK pathway activation [27, 58, 59]. Thus, when tumor cells experience cellular stress and/or when TGFβ1 is abundant in the TME, pS134-GR self-perpetuates its own phosphorylation by inducing the expression of MAP3K5 which in turn activates p38 MAPK in cooperation with 14-3-3ζ. Finally, this signaling axis enables sustained biological responses such as anchorage-independent growth or persistent cell migration of TNBC cells (Fig. 8).

Ser134 phosphorylation is known to promote GR interaction with 14-3-3ζ [15]. A growing body of evidence has implicated 14-3-3ζ in breast cancer progression [60]. The 14-3-3 family of proteins functions as important scaffolds for MAPK modules and numerous other signaling molecules. Relevant to our studies on TNBC cell migration herein, 14-3-3ζ mediates increased invasion of ER-breast cancer cells [61]. We find that like pS134-GR, 14-3-3ζ is required for TGFβ-mediated migration of TNBC models. Interestingly, TNBC cells expressing phospho-mutant S134A-GR, phenocopy wt-GR cells expressing sh14-3-3ζ; cell migration is profoundly impaired upon loss of this MAP3K5-dependent arm of the p38 signaling pathway. Accordingly, 14-3-3ζ has been shown to be important for the activity of MAP3K5 and p38 in other models [62]. Taken together, we conclude that 14-3-3ζ orchestrates p38-dependent phosphorylation of GR Ser134 and interacts with pS134-GR; co-IP assays confirmed regulated GR/14-3-3ζ interaction in TNBC cells (Fig. 4e). The precise role(s) of 14-3-3ζ interaction with pS134-GR is unknown. All SRs rapidly and dynamically shuttle between the cytoplasmic and nuclear compartments. Similar to other SR family members (ER, PR, AR), pS134-GR may interface with rapidly activated cytoplasmic signaling pathways at or near the cell membrane. Via cytoplasmic interaction with 14-3-3ζ, pS134-GR may help nucleate or stabilize functional p38 MAPK modules thereby enabling ultrasensitive signal transduction. Additionally, nuclear 14-3-3ζ proteins may contribute to pS134-GR transcriptional activity or gene promoter selection, perhaps via sustained recruitment of required kinases or other signaling molecules into pS134-GR-containing transcription complexes. These are topics for future study.

The role of posttranslational modifications of GR and their impact on breast cancer prognosis have not yet been elucidated. Herein, we identified genes that are dependent on pS134-GR for their expression; this gene signature may be exploited to more accurately predict which breast cancer patients are more likely to succumb to metastatic disease. For our analysis, we included data for all breast cancer patients from both METABRIC and TCGA. We determined that our 24-gene signature is able to stratify BC patients according to their overall survival. Because we used all breast cancer patients for our analysis, it is possible that pS134-GR has detrimental effects for prognosis in breast cancer patients’ overall (i.e., this observation is not limited to TNBC). ChIP assays (Fig. 7d) show recruitment of GR to TGFβ1-induced genes via pS134-GR, *PIK3IP1*, and *LEFTY2*, in the absence of exogenously added Dex. Future studies aim to confirm genome-wide occupancy of unliganded pS134-GR at novel target genes defined by our signature. Finally, TNBC patients are routinely treated with corticosteroids to alleviate the side effects of chemotherapy. Notably, taxane chemotherapies (e.g., Taxol) activate a cellular stress response that induces robust p38 MAPK activation and sustained GR Ser134 phosphorylation [16]. In future, in addition to standard-of-care approaches, co-targeting pS134-GR and p38 MAPK could provide a better way to limit disease progression in TNBC and other BC subtypes that express pS134-GR.

## Conclusions

We conclude that phospho-GR is a key mediator of dangerous TNBC progression. Herein, we identified that ligand-independent but p38 MAPK-induced phosphorylation of GR on Ser134 is essential for its deleterious actions as a driver of TNBC migration, invasion, anchorage-independent cell growth, and tumorsphere formation. At the molecular level, pS134-GR is required for basal expression of MAP3K5, a required MAP kinase kinase kinase component of the intact p38 MAPK module. With regard to clinical relevance, we identified a pS134-GR 24-gene signature induced by TGFβ1 that may serve as a valuable paired diagnostic with which to identify patients who have GR-driven breast tumors and are thus at high risk of succumbing to metastatic disease. As GR is a ubiquitous steroid hormone receptor, pS134-GR may function similarly in other endocrine-related and/or highly metastatic forms of neoplasia (i.e., prostate cancer, ovarian cancer, melanoma). More work is needed to explore these avenues for clinical translation.

## Additional files

Additional file 1. Expression data 14-3-3ζ (*YWHAZ*).

Additional file 2. Expression data MAP3K5.

Additional file 3. Correlation between GR (*NR3C1*) and MAP3K5.

Additional file 4. Survival data 14-3-3ζ *(YWHAZ).*

Additional file 5. Survival data pS134-GR gene signature in METABRIC.

Additional file 6. Survival data pS134-GR gene signature in TCGA via SurvExpress.

Additional file 7: **Figure S1.** Dexamethasone either inhibits or promotes HS578T breast cancer cell migration in a time-dependent manner. (A) Schematic of protocol used for B and C. Hs578T cells were pretreated with increasing doses of Dex for either 15 mins (B) or 6 hrs (C) and cell migration was analyzed by measuring scratch-wound closure at 18 hrs in the presence of their respective treatments. The mean of three field images from each of the three biological replicates is shown ± SD. Fraction of wound area closure of MDA-MB-231 cells was determined using ImageJ. Statistical significance was assessed by One-way ANOVA and Dunnett’s post-hoc for comparison within groups vs. vehicle treatment (*, *P* < 0.05, **, *P* < 0.01 ****, *P* < 0.0001). (D) Fraction of wound area closure of Hs578T cells treated with vehicle control, TGFβ1 (10 ng/mL), Dex (1μM), TGFβ1+Dex, RU486 (RU; 1μM), RU+TGFβ1 or RU+Dex. The mean of three field images from each of the three biological replicates is shown ± SD. Statistical significance was assessed by One-way ANOVA and Tukey post-hoc for comparison within groups (**, *P* < 0.01, ****, *P* < 0.0001).

Additional file 8: **Figure S2.** Phosphorylation of pS134-GR in TNBC models. (A) Densitometric analysis for pS134-GR levels and p-p38 levels of two independents experiments representative of Figure 2A. Values are relative to the vehicle-control of the wt-GR group and are presented as the mean ± SEM. One-way ANOVA and Fisher’s LSD test posthoc were used to evaluate statistical significance (**, *P* < 0.01,*** *P* < 0.001, ****, *P* < 0.0001). (B) Densitometric analysis for pS134-GR levels and p-p38 levels of two independents experiments representative of Figure 2B. Values are relative to the vehicle-control and are presented as the mean ± SEM. One-way ANOVA and Fisher’s LSD test posthoc were used to evaluate statistical significance (*, *P* < 0.05, **, *P* < 0.01,*** *P* < 0.001, ****, *P* < 0.0001). (C) Densitometric analysis for pS134-GR levels and p-p38 levels of two independents experiments representative of Figure 2C. Values are relative to the vehicle-control and are presented as the mean ± SEM. One-way ANOVA and Fisher’s LSD test posthoc were used to evaluate statistical significance (***, *P* < 0.001). The difference in the levels of p-p38 did not reach statistical significance but an upward trend was observed. (D) Representative Western blot analysis of pS134-GR, total GR, p-p38, and total p38 in MDA-MB-231 cells pre-treated with either 10μM SB203580 (p38 inhibitor) SB203580 (p38 inhibitor), SB202190 (p38 inhibitor), LY294002 (Akt inhibitor), and UO-126 (MEK1/2), or DMSO control for 30 mins followed by either vehicle control or 10 ng/mL of TGF for 1hr. Densitometric analysis is shown with the values of either pS134-GR or p-p38 MAPK relative to vehicle-control of each inhibitor. (E) A similar approach was taken using Hs578T cells. (F) Densitometric analysis for pS134-GR levels and p-p38 levels of two independents experiments (1 hr) representative of Figure 2E. Values are relative to the vehicle-control and are presented as the mean ± SEM. One-way ANOVA and Fisher’s LSD test posthoc were used to evaluate statistical significance (*, *P* < 0.05).

Additional file 9: **Figure S3.** Invasive ability of MDA-MB-231 cells. Cells were plated and allowed to invade through Matrigel transwell for approximately 18 hrs with either vehicle or 10 ng/mL of TGFβ1.

Additional file 10: **Figure S4.** GR regulates the expression of cell movement related pathways. (A) Volcano plot showing differential expression of genes in wt-GR+ and S134A-GR+ TNBC cells treated for 6 hrs with 10 ng/mL of TGFβ1. The number for differentially expressed upregulated genes is included (absolute log2 fold-change of 1 and a p-adj (Benjamini-Hochberg) <0.05). (B) IPA migration-related pathways in wt-GR vs S134A-GR cells treated with TGFβ1 (10 ng/mL); *p*-values and activation z-scores are indicated for each pathway. Genes included for this analysis are based on the following criteria: absolute log2 fold-change of 1.5 and a p-adj (Benjamini-Hochberg) <0.05. (C) Upstream regulators analysis via METACORE of genes that are compared between wt-GR and S134A-GR.

Additional file 11: **Figure S5.** MAP3K5 expression is elevated in TNBC relative to other breast cancer subtypes. (A) Densitometric analysis for pS134-GR levels and p-p38 levels of two independents experiments representative of Figure 6B. Values are relative to the vehicle-control of the wt-GR group and are presented as the mean ± SEM. One-way ANOVA and Fisher’s LSD test posthoc were used to evaluate statistical significance (*, *P* < 0.05, **, *P* < 0.01). (B) Densitometric analysis for pS134-GR levels and p-p38 levels of two independents experiments representative of Figure 6C. Values are relative to the vehicle-control of the shcontrol vehicle group and are presented as the mean ± SEM. One-way ANOVA and Fisher’s LSD test posthoc were used to evaluate statistical significance (*, *P* < 0.05, **, *P* < 0.01). (C) *MAP3K5* mRNA levels were assessed using qRT-PCR following normalization to *TBP* expression; inset shows MAP3K5 protein expression (densitometric levels relative to wt-GR). Mean expression of three independent replicates ± SD is shown. (D) Relative mRNA expression of MAP3K5 in different breast cancer subtypes from the METABRIC cohort (*n*=1700). One-way ANOVA and Tukey post-hoc corrections were used to evaluate statistical significance (****, *P* < 0.0001). (E) pERK1/2 and pJNK levels were assessed as well as total ERK1/2 and JNK levels. Timepoints are shown for 10ng/mL of TGFβ1 treatment. Densitometric levels for pS134-GR are shown relative to vehicle-control. (F) Western blot analysis of pSMAD2 and SMAD2 levels in MDA-MB-231 cells treated with 10ng/mL of TGFβ1. Densitometric values for phospho-SMAD2 are indicated relative to vehicle-control in wt-GR+ cells.

Additional file 12: **Table S1.** Ingenuity Pathway Analysis of GLM approach to compare responsiveness to TGFβ1 for wt-GR and S134A-GR cells. (A) Differentially expressed genes with their respective false discovery rate and log2 fold change as retrieved from our EdgeR analysis for Figure 5B (right). Red rectangles indicates TGFβ1-regulated genes. (B) Genes included for IPA analysis are based on the following criteria: absolute log2 fold-change of 1.0 and a p-adj (Benjamini-Hochberg) <0.05. Because of the limited amount of genes no predictive z-score was reported by IPA.

Additional file 13: **Table S2.** pS134-GR regulates pathways related to cell migration and other advanced cancer behaviors. The top 15 pathways identified in the GSEA analyses for the KEGG molecular signatures are shown with p-values and respective FDR, determined by the R fgsea package.

Additional file 14: **Table S3.** Twenty-four genes are upregulated by TGFβ1-induced pS134-GR. The log2 fold change and p-values and p-adj (Benjamini-Hochberg) associated with the pS134-GR gene-signature are shown in the wt-GR cells.

Additional file 15: **Table S4.** Log2 Fold Change and p-adjusted values for genes illustrated in the heatmap for the wt-GR cells.

Additional file 16: **Table S5.** Material and reagents table.

## Data Availability

The datasets supporting the conclusions of this article are included in the supporting files associated to this article. The expression datasets analyzed in the current study are available in the GEO repository (GSE148444).

## Abbreviations

TNBC: Triple-negative breast cancer
GR: Glucocorticoid receptor
pS134-GR: Phospho-Ser134 Glucocorticoid Receptor
WT: Wild-type
BC: Breast cancer
ER: Estrogen receptor
PR: Progesterone receptor
HER2: Human Epidermal Growth Factor
Dex: Dexamethasone
SR: Steroid Hormone Receptor
HIF2: Hypoxia-Inducible Factor 2
AhR: Aryl Hydrocarbon Receptor
Brk: Breast Tumor Kinase
TME: Tumor microenvironment
TGFβ: Transforming Growth Factor Beta
EMT: Epithelial to mesenchymal transition
STR: Short-tandem repeat
PAM: Protospacer adjacent motif
HGF: Hepatocyte Growth Factor
DMSO: Dimethyl sulfoxide
IMEM: Improved Minimum Essential Media
METABRIC: Molecular Taxonomy of Breast Cancer International Consortium
PCA: Principal component analysis
qRT-PCR: Quantitative real-time PCR
TBP: *TATA-binding protein*
ChIP: Chromatin Immunoprecipitation Assay
FBS: Fetal bovine serum
DCC: Steroid hormone-free fetal bovine serum
GLM: Generalized linear model
IPA: Ingenuity Pathway Analysis
GSEA: Gene Set Enrichment Analysis
KEGG: Kyoto Encyclopedia of Genes and Genomes
GRE: Glucocorticoid response element
MKKK: MAP kinase kinase kinase
