# Supplementary figures and images for: Glucocorticoid receptors are required effectors of TGFβ1-induced p38 MAPK signaling to advanced cancer phenotypes in triple-negative breast cancer
Source: Breast Cancer Res. 2020 May 1;22:39. doi: 10.1186/s13058-020-01277-8 (PMC7193415; doi:10.1186/s13058-020-01277-8)

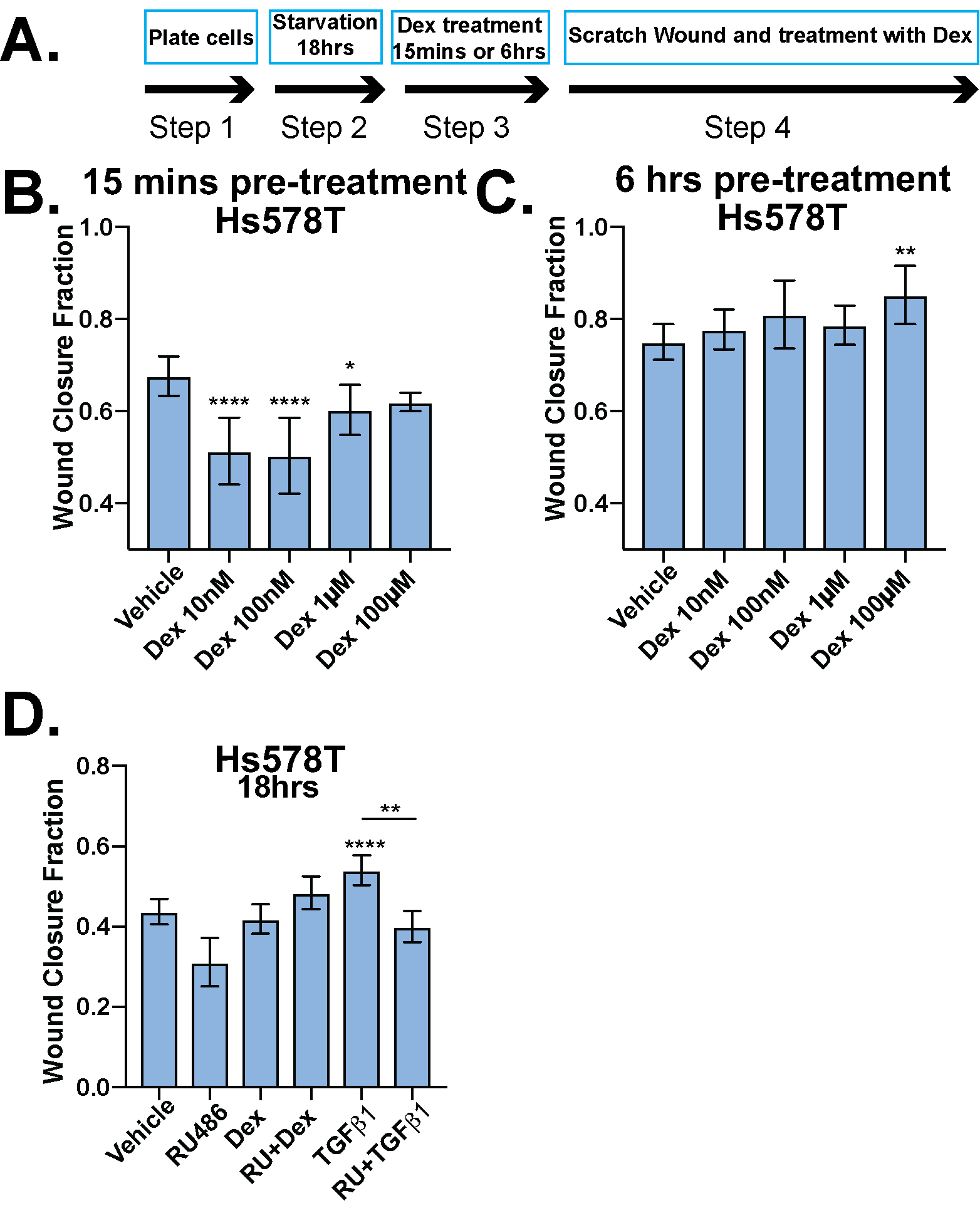

Supplement: Supplementary file 7 — Figure S1. Dexamethasone either inhibits or promotes HS578T breast cancer cell migration in a time-dependent manner. (A) Schematic of protocol used for B and C. Hs578T cells were pretreated with increasing doses of Dex for either 15 mins (B) or 6 hrs (C) and cell migration was analyzed by measuring scratch-wound closure at 18 hrs in the presence of their respective treatments. The mean of three field images from each of the three biological replicates is shown ± SD. Fraction of wound area closure of MDA-MB-231 cells was determined using ImageJ. Statistical significance was assessed by One-way ANOVA and Dunnett’s post-hoc for comparison within groups vs. vehicle treatment (*, P < 0.05, **, P < 0.01 ****, P < 0.0001). (D) Fraction of wound area closure of Hs578T cells treated with vehicle control, TGFβ1 (10 ng/mL), Dex (1μM), TGFβ1+Dex, RU486 (RU; 1μM), RU+TGFβ1 or RU+Dex. The mean of three field images from each of the three biological replicates is shown ± SD. Statistical significance was assessed by One-way ANOVA and Tukey post-hoc for comparison within groups (**, P < 0.01, ****, P < 0.0001). [file 13058_2020_1277_MOESM7_ESM.tif]

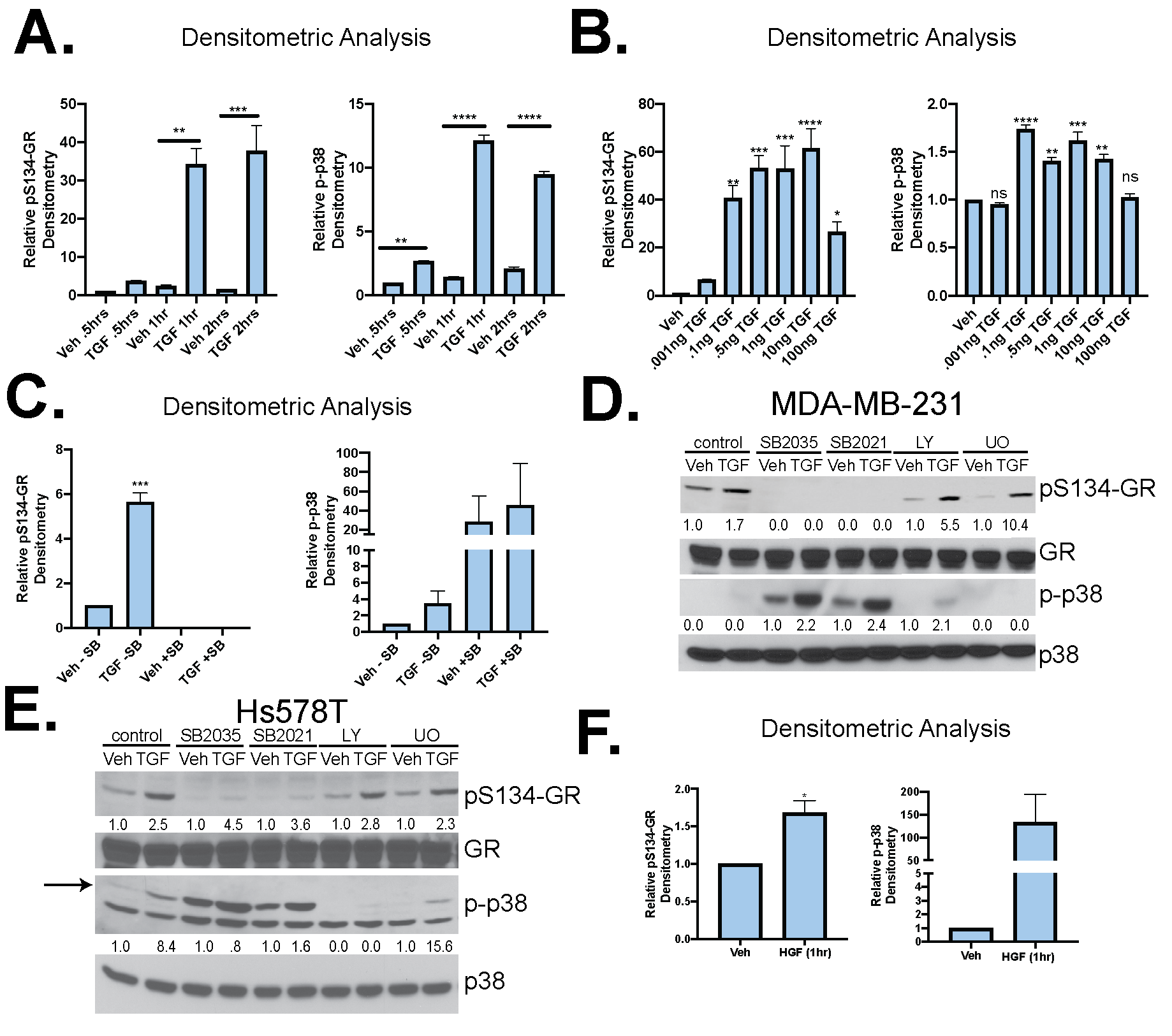

Supplement: Supplementary file 8 — Figure S2. Phosphorylation of pS134-GR in TNBC models. (A) Densitometric analysis for pS134-GR levels and p-p38 levels of two independents experiments representative of Figure 2A. Values are relative to the vehicle-control of the wt-GR group and are presented as the mean ± SEM. One-way ANOVA and Fisher’s LSD test posthoc were used to evaluate statistical significance (**, P < 0.01,*** P < 0.001, ****, P < 0.0001). (B) Densitometric analysis for pS134-GR levels and p-p38 levels of two independents experiments representative of Figure 2B. Values are relative to the vehicle-control and are presented as the mean ± SEM. One-way ANOVA and Fisher’s LSD test posthoc were used to evaluate statistical significance (*, P < 0.05, **, P < 0.01,*** P < 0.001, ****, P < 0.0001). (C) Densitometric analysis for pS134-GR levels and p-p38 levels of two independents experiments representative of Figure 2C. Values are relative to the vehicle-control and are presented as the mean ± SEM. One-way ANOVA and Fisher’s LSD test posthoc were used to evaluate statistical significance (***, P < 0.001). The difference in the levels of p-p38 did not reach statistical significance but an upward trend was observed. (D) Representative Western blot analysis of pS134-GR, total GR, p-p38, and total p38 in MDA-MB-231 cells pre-treated with either 10μM SB203580 (p38 inhibitor) SB203580 (p38 inhibitor), SB202190 (p38 inhibitor), LY294002 (Akt inhibitor), and UO-126 (MEK1/2), or DMSO control for 30 mins followed by either vehicle control or 10 ng/mL of TGF for 1hr. Densitometric analysis is shown with the values of either pS134-GR or p-p38 MAPK relative to vehicle-control of each inhibitor. (E) A similar approach was taken using Hs578T cells. (F) Densitometric analysis for pS134-GR levels and p-p38 levels of two independents experiments (1 hr) representative of Figure 2E. Values are relative to the vehicle-control and are presented as the mean ± SEM. One-way ANOVA and Fisher’s LSD test posthoc were used to e [file 13058_2020_1277_MOESM8_ESM.tif]

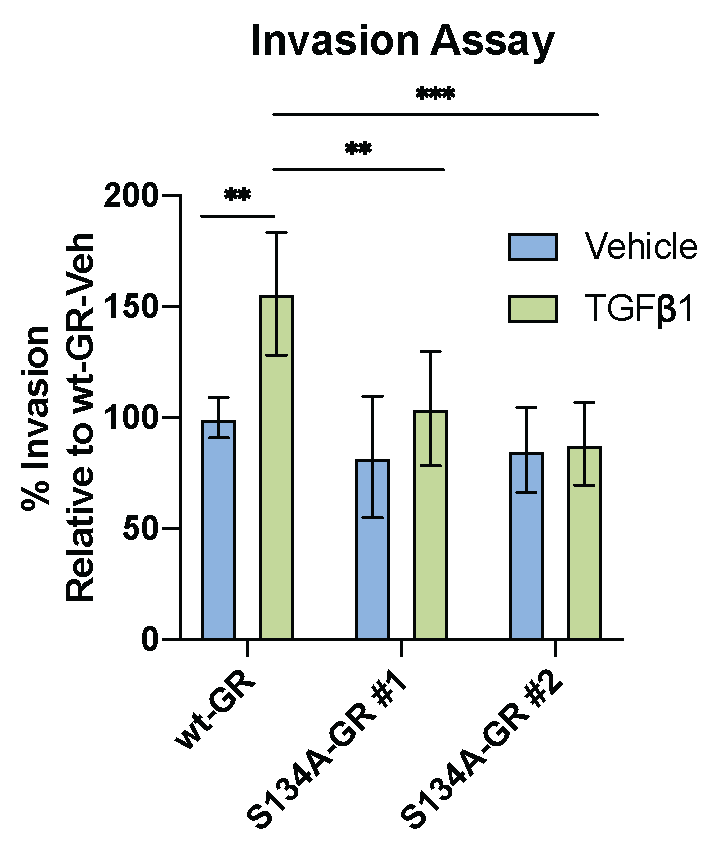

Supplement: Supplementary file 9 — Figure S3. Invasive ability of MDA-MB-231 cells. Cells were plated and allowed to invade through Matrigel transwell for approximately 18 hrs with either vehicle or 10 ng/mL of TGFβ1. [file 13058_2020_1277_MOESM9_ESM.tif]

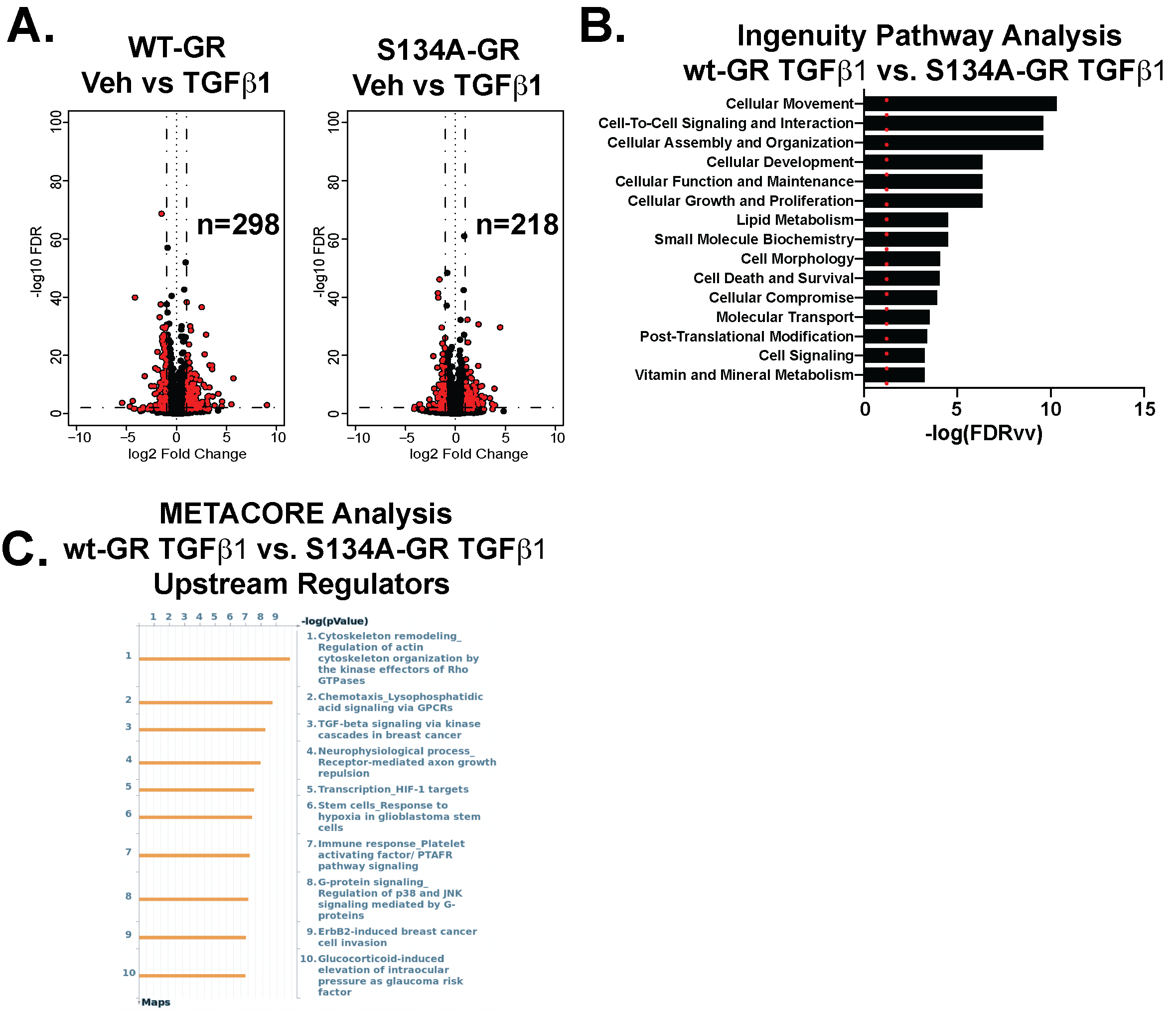

Supplement: Supplementary file 10 — Figure S4. GR regulates the expression of cell movement related pathways. (A) Volcano plot showing differential expression of genes in wt-GR+ and S134A-GR+ TNBC cells treated for 6 hrs with 10 ng/mL of TGFβ1. The number for differentially expressed upregulated genes is included (absolute log2 fold-change of 1 and a p-adj (Benjamini-Hochberg) <0.05). (B) IPA migration-related pathways in wt-GR vs S134A-GR cells treated with TGFβ1 (10 ng/mL); p-values and activation z-scores are indicated for each pathway. Genes included for this analysis are based on the following criteria: absolute log2 fold-change of 1.5 and a p-adj (Benjamini-Hochberg) <0.05. (C) Upstream regulators analysis via METACORE of genes that are compared between wt-GR and S134A-GR. [file 13058_2020_1277_MOESM10_ESM.tif]

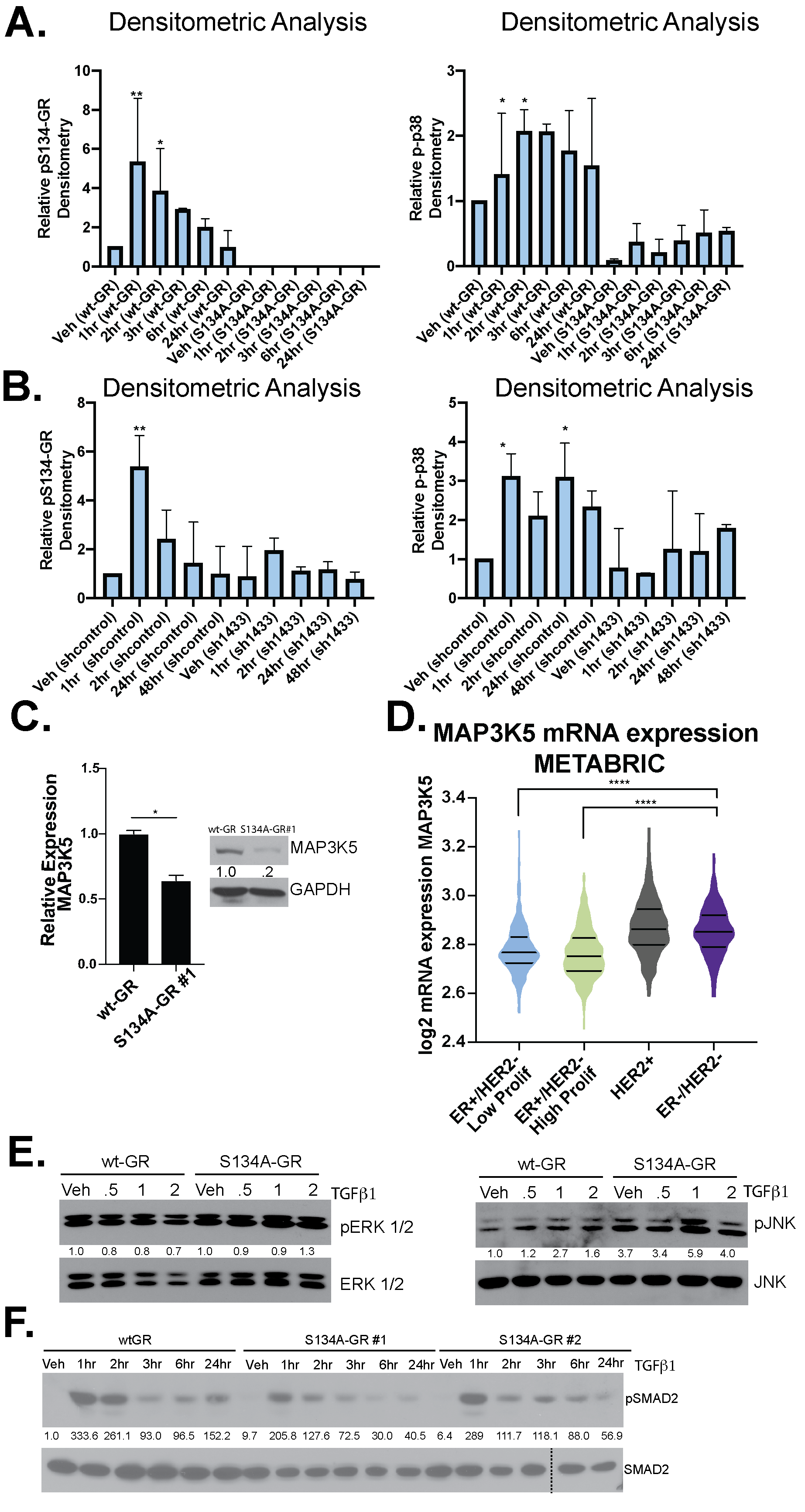

Supplement: Supplementary file 11 — Figure S5. MAP3K5 expression is elevated in TNBC relative to other breast cancer subtypes. (A) Densitometric analysis for pS134-GR levels and p-p38 levels of two independents experiments representative of Figure 6B. Values are relative to the vehicle-control of the wt-GR group and are presented as the mean ± SEM. One-way ANOVA and Fisher’s LSD test posthoc were used to evaluate statistical significance (*, P < 0.05, **, P < 0.01). (B) Densitometric analysis for pS134-GR levels and p-p38 levels of two independents experiments representative of Figure 6C. Values are relative to the vehicle-control of the shcontrol vehicle group and are presented as the mean ± SEM. One-way ANOVA and Fisher’s LSD test posthoc were used to evaluate statistical significance (*, P < 0.05, **, P < 0.01). (C) MAP3K5 mRNA levels were assessed using qRT-PCR following normalization to TBP expression; inset shows MAP3K5 protein expression (densitometric levels relative to wt-GR). Mean expression of three independent replicates ± SD is shown. (D) Relative mRNA expression of MAP3K5 in different breast cancer subtypes from the METABRIC cohort (n=1700). One-way ANOVA and Tukey post-hoc corrections were used to evaluate statistical significance (****, P < 0.0001). (E) pERK1/2 and pJNK levels were assessed as well as total ERK1/2 and JNK levels. Timepoints are shown for 10ng/mL of TGFβ1 treatment. Densitometric levels for pS134-GR are shown relative to vehicle-control. (F) Western blot analysis of pSMAD2 and SMAD2 levels in MDA-MB-231 cells treated with 10ng/mL of TGFβ1. Densitometric values for phospho-SMAD2 are indicated relative to vehicle-control in wt-GR+ cells. [file 13058_2020_1277_MOESM11_ESM.tif]

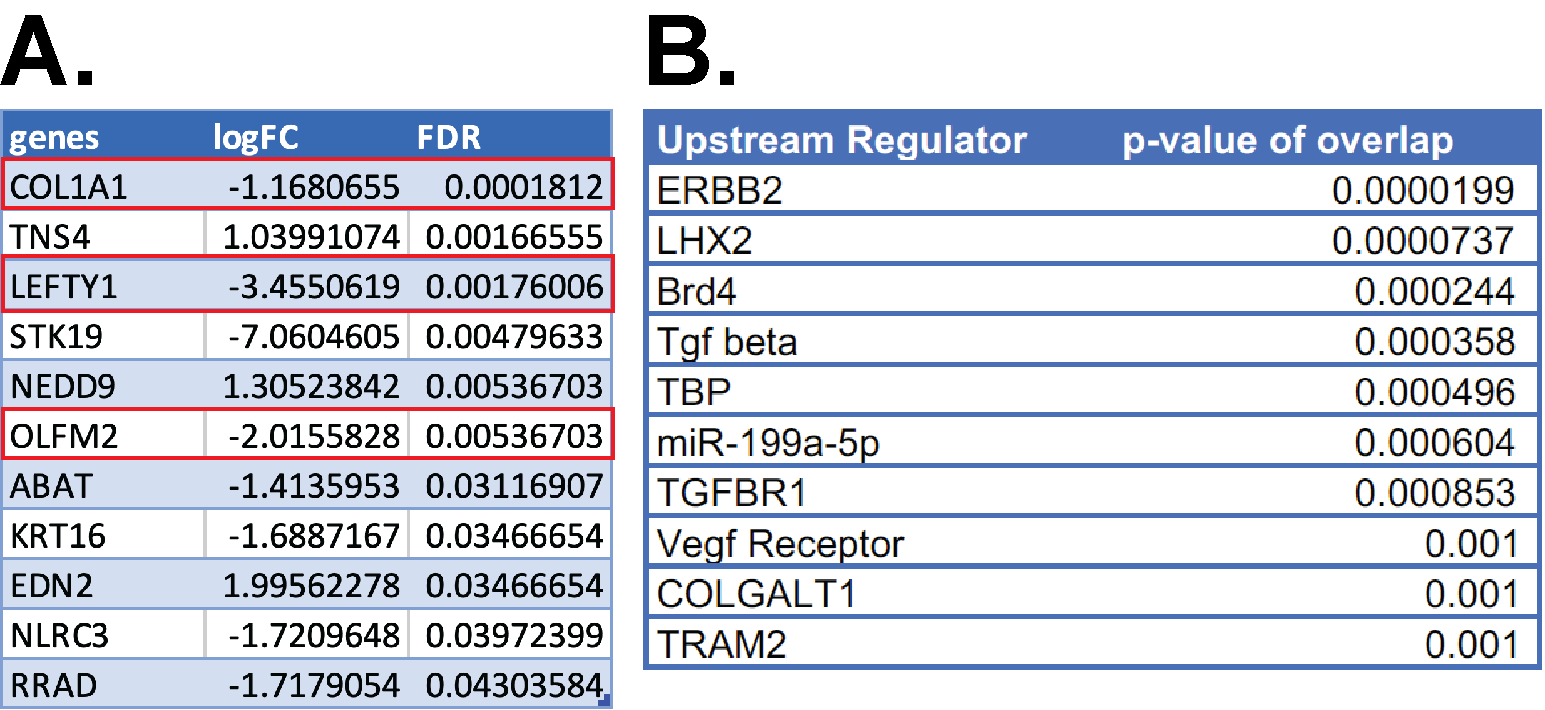

Supplement: Supplementary file 12 — Table S1. Ingenuity Pathway Analysis of GLM approach to compare responsiveness to TGFβ1 for wt-GR and S134A-GR cells. (A) Differentially expressed genes with their respective false discovery rate and log2 fold change as retrieved from our EdgeR analysis for Figure 5B (right). Red rectangles indicates TGFβ1-regulated genes. (B) Genes included for IPA analysis are based on the following criteria: absolute log2 fold-change of 1.0 and a p-adj (Benjamini-Hochberg) <0.05. Because of the limited amount of genes no predictive z-score was reported by IPA. [file 13058_2020_1277_MOESM12_ESM.tif]

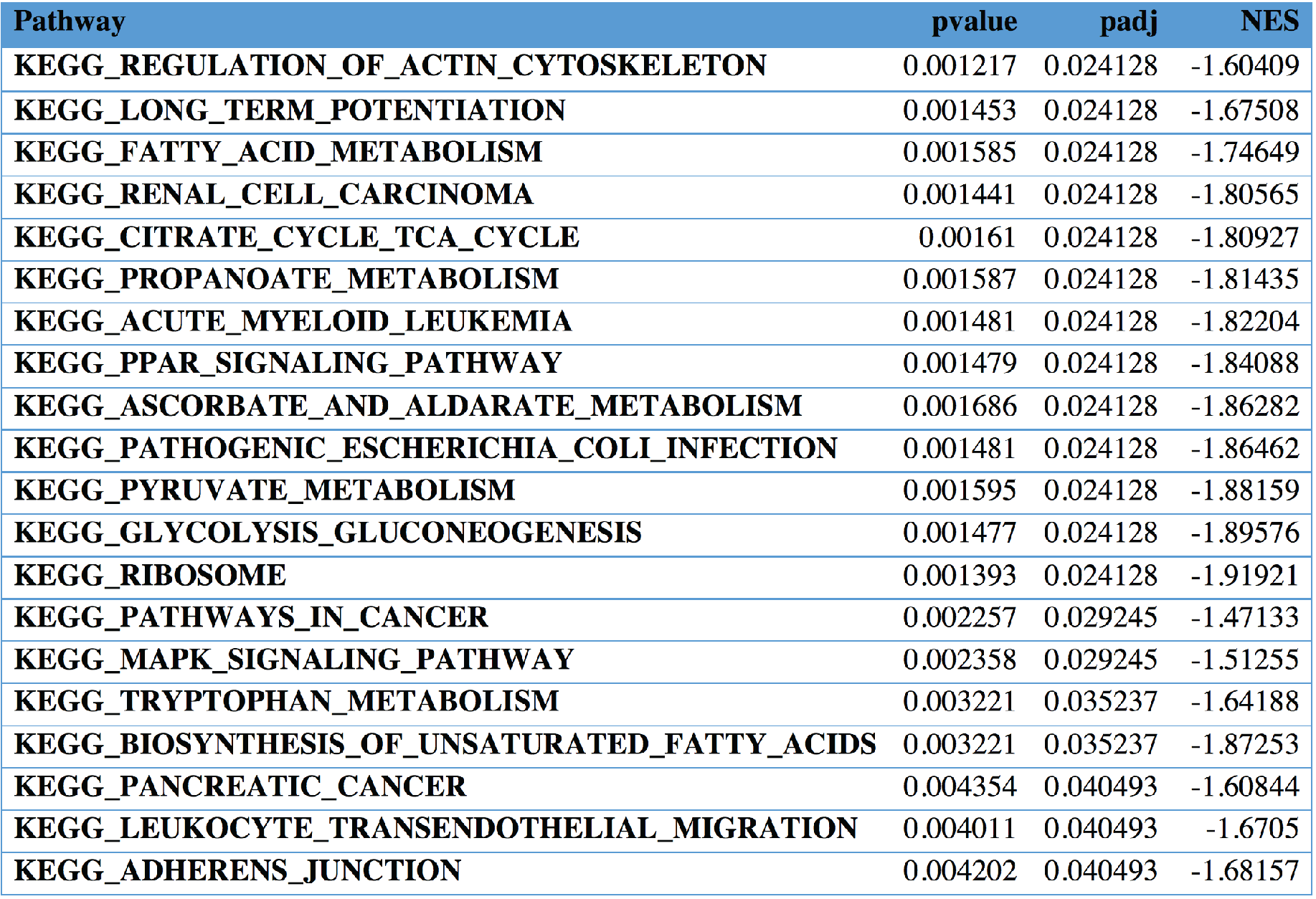

Supplement: Supplementary file 13 — Table S2. pS134-GR regulates pathways related to cell migration and other advanced cancer behaviors. The top 15 pathways identified in the GSEA analyses for the KEGG molecular signatures are shown with p-values and respective FDR, determined by the R fgsea package. [file 13058_2020_1277_MOESM13_ESM.tif]

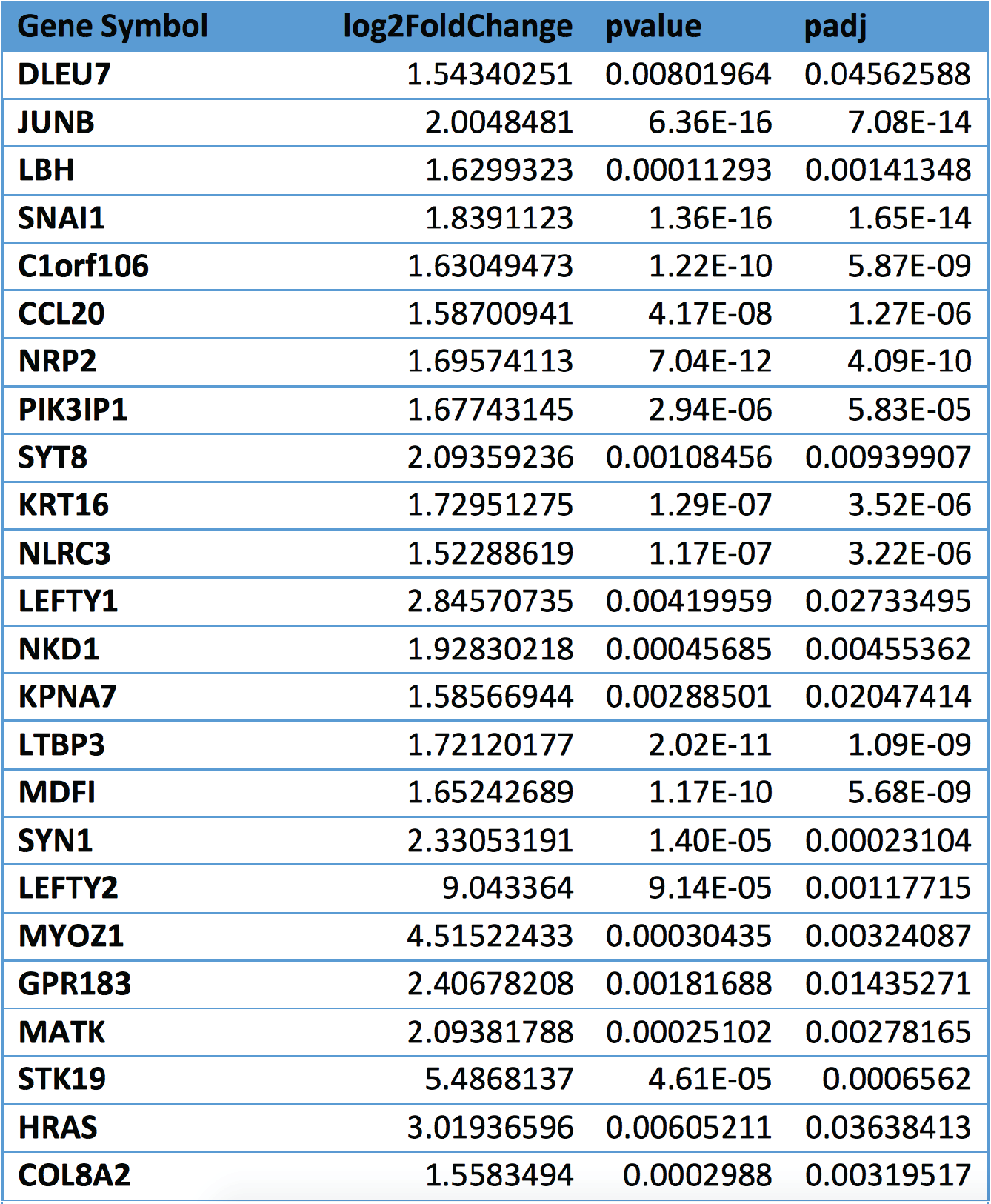

Supplement: Supplementary file 14 — Table S3. Twenty-four genes are upregulated by TGFβ1-induced pS134-GR. The log2 fold change and p-values and p-adj (Benjamini-Hochberg) associated with the pS134-GR gene-signature are shown in the wt-GR cells. [file 13058_2020_1277_MOESM14_ESM.tif]

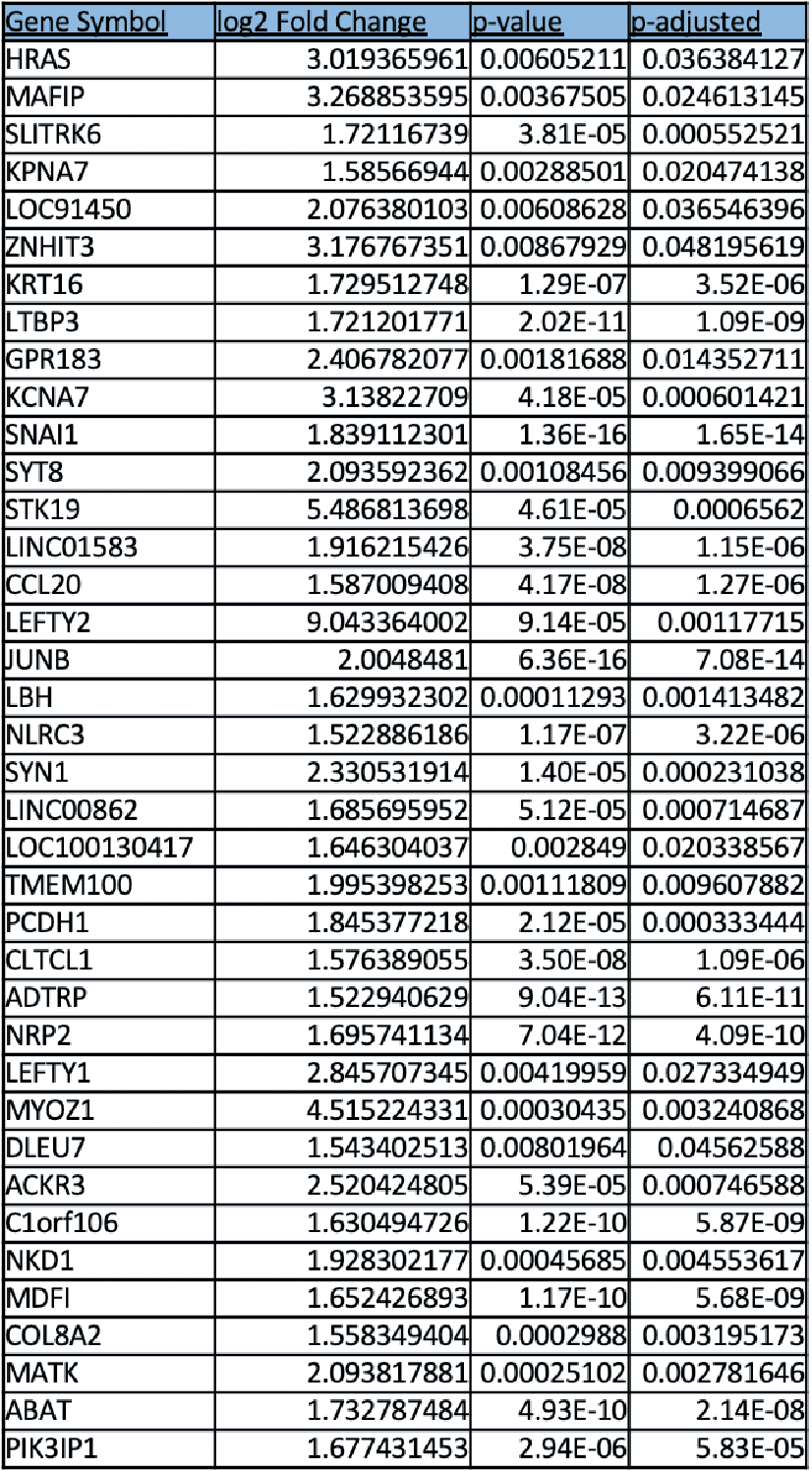

Supplement: Supplementary file 15 — Table S4. Log2 Fold Change and p-adjusted values for genes illustrated in the heatmap for the wt-GR cells. [file 13058_2020_1277_MOESM15_ESM.tif]
